# Supplementary material for: The effectiveness of case management interventions for the homeless, vulnerably housed and persons with lived experience: A systematic review
Source: PLoS One. 2020 Apr 9;15(4):e0230896. doi: 10.1371/journal.pone.0230896 (PMC7313544; doi:10.1371/journal.pone.0230896)
Supplement: S2 File — (PDF) [file pone.0230896.s002.pdf]

The Effectiveness of Case-Management Interventions for the Homeless, Vulnerably Housed and Persons with Lived Experience: A Systematic Review and Meta-Analysis.

Appendix II: Sample search strategy

1 vulnerable populations/ poverty areas/

2 ((deprived or destitute? or impoverished or low income or marginalized or marginalized or needy or poverty or vulnerable) adj2 (adolesc$ or child$ or famil$ or men or people or youth? or women)).tw,kf.

3 homeless persons/ homeless youth/ runaway behavior/

4 (homeless$ or runaway?).tw,kf.

5 (temporar$ adj2 (accommodat$ or home? or hous$)).tw,kf.

6 ((based or housed or residen$ or temporar$) adj2 shelter?).tw,kf.

7 or/1-7

8 exp program evaluation/

9 (effectiveness or initiative? or prevent$ or program$ or reduc$ or strateg$ or treatment).tw.

10 or/8-9

11 systematic review/ meta-analysis/ randomized controlled trial/ controlled clinical trial/ pragmatic clinical trial/ controlled before-after studies/ interrupted time series analysis/ controlled before-after studies/ (randomized or randomized).ab,kf.

12 (before adj2 after adj5 (design$ or study or trial)).tw,kf.

13 ((preintervention? or pre-intervention? or postintervention? or post intervention?) adj5 (study or trial)).tw,kf.

14 ((pre-test or pretest or (posttest or post test)) adj2 (design$ or method$ or study or trial)).tw,kf.

15 *economics/ exp *"Costs and Cost Analysis"/ economics, nursing/ economics, medical/ economics, pharmaceutical/ exp economics, hospital/ economics, dental/ exp "Fees and Charges"/ exp budgets/

16 ((budget$ or economic$ or cost or costs or costly or costing or price or prices or pricing or pharmacoeconomic$ or pharmaco-economic$ or expenditure or expenditures or expense or expenses or financial or finance or finances or financed) adj6 (analys$ or analyz$ or effect$ or evaluat$ or impact$)).ab. /freq=2

17 (cost$ adj2 (effective$ or utilit$ or benefit$ or minimi$ or analy$ or outcome or outcomes)).ab,kf.

18 (value adj2 (money or monetary)).tw,kf.

19 exp models, economic/ economic model$.ab,kf.
