# Supplementary material for: The effectiveness of case management interventions for the homeless, vulnerably housed and persons with lived experience: A systematic review
Source: PLoS One. 2020 Apr 9;15(4):e0230896. doi: 10.1371/journal.pone.0230896 (PMC7313544; doi:10.1371/journal.pone.0230896)
Supplement: S3 File — (PDF) [file pone.0230896.s003.pdf]

The Effectiveness of Case-Management Interventions for the Homeless, Vulnerably Housed and Persons with Lived Experience: A Systematic Review and Meta-Analysis.

Appendix III: List of Excluded Studies

#	Standard Case management studies	Reason for exclusion
1	Nyamathi A, Salem BE, Zhang S, Farabee D, Hall B, Khalilifard F, Leake B. Nursing case management, peer coaching, and hepatitis a and B vaccine completion among homeless men recently released on parole: randomized clinical trial. Nursing research. 2015 May;64(3):177.	Wrong study design
2	Slesnick N, Erdem G. Intervention for homeless, substance abusing mothers: Findings from a non-randomized pilot. Behavioral Medicine. 2012 Apr 1;38(2):36-48.	Wrong study design
3	Althaus F, Paroz S, Hugli O, Ghali WA, Daeppen JB, Peytremann-Bridevaux I, Bodenmann P. Effectiveness of interventions targeting frequent users of emergency departments: a systematic review. Annals of emergency medicine. 2011 Jul 1;58(1):41-52.	Wrong study design
4	Fitzpatrick-Lewis D, Ganann R, Krishnaratne S, Ciliska D, Kouyoumdjian F, Hwang SW. Effectiveness of interventions to improve the health and housing status of homeless people: a rapid systematic review. BMC public health. 2011 Dec;11(1):638.	Wrong study design
5	de Vet R, van Luijtelaar MJ, Brilleslijper-Kater SN, Vanderplasschen W, Beijersbergen MD, Wolf JR. Effectiveness of case management for homeless persons: a systematic review. American Journal of Public Health. 2013 Oct;103(10):e13-26.	Wrong study design
6	Kumar GS, Klein R. Effectiveness of case management strategies in reducing emergency department visits in frequent user patient populations: a systematic review. The Journal of emergency medicine. 2013 Mar 1;44(3):717-29.	Wrong study design
7	Zlotnick C, Tam T, Zerger S. Common needs but divergent interventions for US homeless and foster care children: Results from a systematic review. Health & Social Care in the Community. 2012 Sep;20(5):449-76.	Wrong study design
8	Hwang SW, Tolomiczenko G, Kouyoumdjian FG, Garner RE. Interventions to improve the health of the homeless: a systematic review. American journal of preventive medicine. 2005 Nov 1;29(4):311-.	Wrong study design
9	Health Quality Ontario. Interventions to improve access to primary care for people who are homeless: A systematic review. Ontario health technology assessment series. 2016;16(9):1.	Wrong study design
10	Luchenski S, Maguire N, Aldridge RW, Hayward A, Story A, Perri P, Withers J, Clint S, Fitzpatrick S, Hewett N. What works in inclusion health: overview of effective interventions for marginalised and excluded populations. The Lancet. 2018 Jan 20;391(10117):266-80.	Wrong study design
11	Hwang SW, Burns T. Health interventions for people who are homeless. The Lancet. 2014 Oct 25;384(9953):1541-7.	Wrong study design
12	Vanderplasschen W, Wolf J, Rapp RC, Broekaert E. Effectiveness of different models of case management for substance-abusing populations. Journal of psychoactive drugs. 2007 Mar 1;39(1):81-95.	Wrong study design
13	Nugent WR, Bruley C, Allen P. The effects of aggression replacement training on antisocial behavior in a runaway shelter. Research on Social Work Practice. 1998 Nov;8(6):637-56.	Wrong study design
14	Sacks JY, Sacks S, Harle M, Leon GD. Homelessness prevention therapeutic community (TC) for addicted mothers. Alcoholism Treatment Quarterly. 1999 Aug 12;17(1-2):33-51.	Wrong study design

15	Nyamathi AM, E. Salem B, Farabee D, Hall E, Zhang S, Marfisee M, Khalilifard F, Musto S, Faucette M, Leake B. Correlates of heroin and methamphetamine use among homeless male ex-jail and prison offenders. Addiction research & theory. 2014 Dec 1;22(6):463-73.	Wrong study design
16	Allen T. The health impact of floating support. Housing, Care and Support. 2006 Dec 1;9(3):6-8.	Wrong study design
17	Yamin S, Aubry T, Volk J, Jetté J, Bourque J, Crouse S. Peer supportive housing for consumers of Housing First who experience ongoing housing instability. Canadian Journal of Community Mental Health. 2015 Apr 24;33(4):61-76.	Wrong study design
18	Collard CS, Lewinson T, Watkins K. Supportive housing: an evidence-based intervention for reducing relapse among low income adults in addiction recovery. Journal of evidence-based social work. 2014 Oct 20;11(5):468-79.	Wrong study design
19	Broner N, Lang M, Behler SA. The effect of homelessness, housing type, functioning, and community reintegration supports on mental health court completion and recidivism. Journal of dual diagnosis. 2009 Nov 24;5(3-4):323-56.	Wrong study design
20	CADTH. Case management for vulnerable or homeless persons: clinical and cost-effectiveness, and guidelines (structured abstract). 2014 Mar; Project Number RA0670-000	Wrong study design
21	Buchanan D, Kee R, Sadowski LS, Garcia D. The health impact of supportive housing for HIV-positive homeless patients: a randomized controlled trial. American journal of public health. 2009 Nov;99(S3):S675-80.	Irrelevant outcomes
22	Kangovi S, Mitra N, Grande D, Huo H, Smith RA, Long JA. Community health worker support for disadvantaged patients with multiple chronic diseases: a randomized clinical trial. American journal of public health. 2017 Oct;107(10):1660-7.	Wrong population
23	Sokol R, Fisher E. Peer support for the hardly reached: a systematic review. American journal of public health. 2016 Jul;106(7):e1-8.	Wrong population
24	Goodson BD, Layzer JI, Pierre RG, Bernstein LS, Lopez M. Effectiveness of a comprehensive, five-year family support program for low-income children and their families: Findings from the Comprehensive Child Development Program. Early Childhood Research Quarterly. 2000 Jan 1;15(1):5-39.	Wrong population
25	Rosenblum A, Magura S, Fong C, Curry P, Norwood C, Casella D. Effects of peer mentoring on HIV-affected youths' substance use risk and association with substance using friends. Journal of social service research. 2006 May 10;32(2):45-60.	Wrong population
26	Erkel EA, Morgan EP, Staples MA, Assey VH, Michel Y. Case management and preventive services among infants from low-income families. Public health nursing. 1994 Oct;11(5):352-60.	Wrong population
27	Felton CJ, Stastny P, Shern DL, Blanch A, Donahue SA, Knight E, Brown C. Consumers as peer specialists on intensive case management teams: impact on client outcomes. Psychiatric Services. 1995 Oct.	Wrong population
28	Trauma-informed temporary assistance for needy families (tanf): A randomized controlled trial with a two-generation impact 2018 Booshehri, Layla G. and Dugan, Jerome and Patel, Falguni and Bloom, Sandra and Chilton, Mariana	Wrong population
29	Needels K, James-Burdumy S, Burghardt J. Community case management for former jail inmates: its impacts on rearrest, drug use, and HIV risk. Journal of Urban Health. 2005 Sep 1;82(3):420-33.	Wrong population
30	Gensichen J, Beyer M, Muth C, Gerlach FM, Von Korff M, Ormel J. Case management to improve major depression in primary health care: a systematic review. Psychological medicine. 2006 Jan;36(1):7-14.	Wrong population
31	Hultman CI, Conrad KJ, Pope AR, Baxter WC, Lisiecki J, Elbaum P. Assessing the	Could not be retrieved

	implementation of a case management intervention for the homeless. Advances in Medical Sociology. 1995;6:295-324.	
32	Stahler G, Shipley Jr TE, Bartelt D, Ducette J, Shandler IW. Evaluating alternative treatments for homeless substance-abusing men: outcomes and predictors of success. Journal of Addictive Diseases. 1996 Jan 10;14(4):151-67.	Wrong intervention
33	Greeson JK, Garcia AR, Kim M, Thompson AE, Courtney ME. Development & maintenance of social support among aged out foster youth who received independent living services: Results from the Multi-Site Evaluation of Foster Youth Programs. Children and youth services review. 2015 Jun 1;53:1-9.	Wrong intervention
34	Pope AR, Conrad KJ, Baxter W, Elbaum P, Lisiecki J, Daghestani A, Hultman C, Lyons J. Case managed residential care for homeless addicted veterans: Evanston/VA. Alcoholism Treatment Quarterly. 1993 Nov 8;10(3-4):155-69.	Wrong intervention
35	Nossel IR, Lee RJ, Isaacs A, Herman DB, Marcus SM, Essock SM. Use of peer staff in a critical time intervention for frequent users of a psychiatric emergency room. Psychiatric Services. 2016 Apr 18;67(5):479-81.	Wrong intervention
36	Rich AR, Clark C. Gender differences in response to homelessness services. Evaluation and program planning. 2005 Feb 1;28(1):69-81.	Wrong intervention
37	Devine JA, Wright JD, Brody CJ. An evaluation of an alcohol and drug treatment program for homeless substance abusers. Evaluation Review. 1995 Dec;19(6):620-45.	Wrong intervention
38	Karper L, Kaufmann M, Millspaugh G, Vega E, Stern G, Stern G, Ezrow DJ, Giansante S, Lynch M. Coordination of care for homeless individuals with comorbid severe mental disorders and substance-related disorders. Journal of Dual Diagnosis. 2008 Jun 10;4(2):142-57.	Wrong intervention
39	Marshall M, Lockwood A, Gath D. Social services case-management for long-term mental disorders: a randomised controlled trial. The Lancet. 1995 Feb 18;345(8947):409-12.	Wrong intervention
40	Basu A, Kee R, Buchanan D, Sadowski LS. Comparative cost analysis of housing and case management program for chronically ill homeless adults compared to usual care. Health services research. 2012 Feb;47(1pt2):523-43.	Wrong intervention
41	Grace M, Gill PR. Improving outcomes for unemployed and homeless young people: Findings of the YP4 clinical controlled trial of joined up case management. Australian Social Work. 2014 Jul 3;67(3):419-37.	Wrong intervention
42	Corrigan PW, Kraus DJ, Pickett SA, Schmidt A, Stellon E, Hantke E, Lara JL. Using peer navigators to address the integrated health care needs of homeless African Americans with serious mental illness. Psychiatric services. 2017 Jan 17;68(3):264-70.	Wrong intervention
43	Nyamathi A, Salem BE, Farabee D, Hall E, Zhang S, Faucette M, Bond D, Yadav K. Impact of an intervention for recently released homeless offenders on self-reported re-arrest at 6 and 12 months. Journal of addictive diseases. 2017 Jan 2;36(1):60-71.	Wrong intervention
44	Barker SL, Maguire N. Experts by experience: peer support and its use with the homeless. Community mental health journal. 2017 Jul 1;53(5):598-612.	Wrong intervention
45	Slesnick N, Erdem G. Efficacy of ecologically-based treatment with substance-abusing homeless mothers: Substance use and housing outcomes. Journal of substance abuse treatment. 2013 Nov 1;45(5):416-25.	Wrong intervention
46	Wood PA, Hurlburt MS, Hough RL, Hofstetter CR. Longitudinal assessment of family support among homeless mentally ill participants in a supported housing program. Journal of Community Psychology. 1998 Jul;26(4):327-44.	Wrong intervention

47	Benston EA. Housing programs for homeless individuals with mental illness: Effects on housing and mental health outcomes. Psychiatric Services. 2015 Apr 15;66(8):806-16.	Wrong intervention
48	Sadowski LS, Kee RA, VanderWeele TJ, Buchanan D. Effect of a housing and case management program on emergency department visits and hospitalizations among chronically ill homeless adults: a randomized trial. Jama. 2009 May 6;301(17):1771-8.	Wrong intervention
49	Lam JA, Jekel JF, Thompson KR, Leaf PJ, Hartwell SW, Florio L. Assessing the value of a short-term residential drug treatment program for homeless men. Journal of Addictive Diseases. 1996 Jan 10;14(4):21-39.	Wrong intervention
50	Gulcur L, Stefancic A, Shinn M, Tsemberis S, Fischer SN. Housing, hospitalization, and cost outcomes for homeless individuals with psychiatric disabilities participating in continuum of care and housing first programmes. Journal of Community & Applied Social Psychology. 2003 Mar 1;13(2):171-86.	Wrong intervention
51	Bybee D, Sullivan CM. Predicting re-victimization of battered women 3 years after exiting a shelter program. American journal of community psychology. 2005 Sep 1;36(1-2):85-96.	Wrong intervention
52	Rosenblum A, Magura S, Kayman DJ, Fong C. Motivationally enhanced group counseling for substance users in a soup kitchen: A randomized clinical trial. Drug and Alcohol Dependence. 2005 Oct 1;80(1):91-103.	Wrong intervention
53	LePage JP, Garcia-Rea EA. Lifestyle coaching's effect on 6-month follow-up in recently homeless substance dependent veterans: A randomized study. Psychiatric Rehabilitation Journal. 2012 Sep;35(5):396.	Wrong intervention
54	Kertesz SG, Larson MJ, Cheng DM, Tucker JA, Winter M, Mullins A, Saitz R, Samet JH. Need and non-need factors associated with addiction treatment utilization in a cohort of homeless and housed urban poor. Medical care. 2006 Mar 1:225-33.	Wrong intervention
55	Fowler PJ, Schoeny M. The Family Unification Program: A randomized-controlled trial of housing stability. Child welfare. 2015;94(1):167.	Wrong intervention
56	LePage JP, Bluitt M, McAdams H, Merrell C, House-Hatfield T, Garcia-Rea E. Effects of increased social support and lifestyle behaviors in a domiciliary for homeless veterans. Psychological Services. 2006 Feb;3(1):16.	Wrong intervention
57	Sajatovic M, Levin J, Ramirez LF, Hahn DY, Tatsuoka C, Bialko CS, Cassidy KA, Fuentes-Casiano E, Williams TD. A prospective trial of customized adherence enhancement plus long-acting injectable antipsychotic medication in homeless or recently homeless individuals with schizophrenia or schizoaffective disorder. The Journal of clinical psychiatry. 2013 Dec;74(12):1249.	Wrong intervention
58	Kneipp SM, Kairalla JA, Lutz BJ, Pereira D, Hall AG, Flocks J, Beeber L, Schwartz T. Public health nursing case management for women receiving temporary assistance for needy families: a randomized controlled trial using community-based participatory research. American journal of public health. 2011 Sep;101(9):1759-68.	Wrong intervention
59	Essock SM, Mueser KT, Drake RE, Covell NH, McHugo GJ, Frisman LK, Kontos NJ, Jackson CT, Townsend F, Swain K. Comparison of ACT and standard case management for delivering integrated treatment for co-occurring disorders. Psychiatric Services. 2006 Feb;57(2):185-96.	Wrong intervention
60	Rosenheck R, Kasprow W, Frisman L, Liu-Mares W. Cost-effectiveness of supported housing for homeless persons with mental illness. Archives of General Psychiatry. 2003 Sep 1;60(9):940-51.	Wrong intervention
61	Okin RL, Boccellari A, Azocar F, Shumway M, O'brien K, Gelb A, Kohn M, Harding P, Wachsmuth C. The effects of clinical case management on hospital service use among ED frequent users. The American journal of emergency medicine. 2000 Sep 1;18(5):603-8.	Wrong intervention

62	Calsyn RJ, Morse GA, Klinkenberg WD, Trusty ML, Allen G. The impact of assertive community treatment on the social relationships of people who are homeless and mentally ill. Community Mental Health Journal. 1998 Dec 1;34(6):579-93.	Wrong intervention
63	Drake RE, Yovetich NA, Bebout RR, Harris M, McHugo GJ. Integrated treatment for dually diagnosed homeless adults. The Journal of Nervous and Mental Disease. 1997 May 1;185(5):298-305.	Wrong intervention
64	Korr WS, Joseph A. Housing the homeless mentally ill: Findings from Chicago. Journal of social service research. 1996 Feb 6;21(1):53-68.	Wrong intervention
65	Haskett ME, Okoniewski KC, Armstrong JM, Galanti S, Lowder E, Loehman J, Lanier PJ. Feasibility, acceptability, and effects of a peer support group to prevent child maltreatment among parents experiencing homelessness. Children and youth services review. 2017 Feb 1;73:187-96.	Wrong intervention
66	Carrasco JM, Fox RA. VARYING TREATMENT INTENSITY IN A HOME-BASED PARENT AND CHILD THERAPY PROGRAM FOR FAMILIES LIVING IN POVERTY: A RANDOMIZED CLINIC TRIAL. Journal of Community Psychology. 2012 Jul;40(5):621-30.	Wrong intervention
67	Lipton FR, Nutt S, Sabatini A. Housing the homeless mentally ill: A longitudinal study of a treatment approach. Psychiatric Services. 1988 Jan;39(1):40-5.	Wrong intervention

#	More intensive case management studies	Reason for exclusion
1	Muser E, Kozma CM, Benson CJ, Mao L, Starr HL, Alphs L, Fastenau J. Cost effectiveness of paliperidone palmitate versus oral antipsychotics in patients with schizophrenia and a history of criminal justice involvement. Journal of medical economics. 2015 Aug 3;18(8):637-45.	Wrong study design
2	Sajatovic M, Ramirez LF, Fuentes-Casiano E, Cage J, Tatsuoka C, Aebi ME, Bukach A, Cassidy KA, Levin JB. A 6-Month Prospective Trial of a Personalized Behavioral Intervention + Long-Acting Injectable Antipsychotic in Individuals With Schizophrenia at Risk of Treatment Nonadherence and Homelessness. Journal of clinical psychopharmacology. 2017 Dec;37(6):702-7.	Wrong study design
3	Chinman MJ, Rosenheck R, Lam JA, Davidson L. Comparing consumer and non consumer provided case management services for homeless persons with serious mental illness. The Journal of nervous and mental disease. 2000 Jul 1;188(7):446-53.	Wrong study design
4	Cooper RL, Seiters J, Davidson DL, MacMaster SA, Rasch RF, Adams S, Darby K. Outcomes of integrated assertive community treatment for homeless consumers with co-occurring disorders. Journal of Dual Diagnosis. 2010 May 7;6(2):152-70.	Wrong study design
5	McGrew JH, Danner M. Evaluation of an intensive case management program for transition age youth and its transition to assertive community treatment. American Journal of Psychiatric Rehabilitation. 2009 Aug 31;12(3):278-94.	Wrong study design
6	Hampton MD, Chafetz L. Factors associated with residential placement in an assertive community treatment program. Issues in Mental Health Nursing. 2002 Jan 1;23(7):677-89.	Wrong study design
7	Draine J, Solomon P. Jail recidivism and the intensity of case management services among homeless persons with mental illness leaving jail. The Journal of Psychiatry & Law. 1994 Jun;22(2):245-61.	Wrong study design
8	Herman DB. Transitional support for adults with severe mental illness: Critical time intervention and its roots in assertive community treatment. Research on Social Work Practice. 2014 Sep;24(5):556-63.	Wrong study design

9	Herman DB, Mandiberg JM. Critical time intervention: Model description and implications for the significance of timing in social work interventions. Research on Social Work Practice. 2010 Sep;20(5):502-8.	Wrong study design
10	Dickey B. Review of programs for persons who are homeless and mentally ill. Harvard review of Psychiatry. 2000 Nov 1;8(5):242-50.	Wrong study design
11	Aubry T, Nelson G, Tsemberis S. Housing first for people with severe mental illness who are homeless: a review of the research and findings from the at home—chez soi demonstration project. The Canadian Journal of Psychiatry. 2015 Nov;60(11):467-74.	Wrong study design
12	Nelson G, Stefancic A, Rae J, Townley G, Tsemberis S, Macnaughton E, Aubry T, Distasio J, Hurtubise R, Patterson M, Stergiopoulos V. Early implementation evaluation of a multi-site housing first intervention for homeless people with mental illness: a mixed methods approach. Evaluation and Program Planning. 2014 Apr 1;43:16-26.	Wrong study design
13	Sajatovic M, Levin J, Ramirez LF, Hahn DY, Tatsuoka C, Bialko CS, Cassidy KA, Fuentes-Casiano E, Williams TD. A prospective trial of customized adherence enhancement plus long-acting injectable antipsychotic medication in homeless or recently homeless individuals with schizophrenia or schizoaffective disorder. The Journal of clinical psychiatry. 2013 Dec;74(12):1249.	Wrong study design
14	Bearman D, Claydon K, Kincheloe J, Lodise C. Breaking the cycle of dependency: dual diagnosis and AFDC families. Journal of psychoactive drugs. 1997 Dec 1;29(4):359-67.	Wrong study design
15	Shern DL, Felton CJ, Hough RL, Lehman AF, Goldfinger S, Valencia E, Dennis D, Straw R, Wood PA. Housing outcomes for homeless adults with mental illness: Results from the second-round McKinney program. Psychiatric services. 1997 Feb 1;48(2):239-41.	Wrong study design
16	Stahler GJ, Shipley TE, Bartelt D, Westcott D, Griffith E, Shandler I. Retention issues in treating homeless polydrug users: Philadelphia. Alcoholism Treatment Quarterly. 1993 Nov 8;10(3-4):201-15.	Wrong study design
17	Dieterich M, Irving CB, Bergman H, Khokhar MA, Park B, Marshall M. Intensive case management for severe mental illness. Cochrane database of systematic reviews. 2017(1).	Wrong study design
18	Hunt GE, Siegfried N, Morley K, Sitharthan T, Cleary M. Psychosocial interventions for people with both severe mental illness and substance misuse. Cochrane Database of Systematic Reviews. 2013(10).	Wrong study design
19	Larsen M, Nordentoft M. Evidence-based treatment of mentally ill homeless persons. Ugeskrift for laeger. 2010 May;172(22):1669-75.	Wrong study design
20	Benston EA. Housing programs for homeless individuals with mental illness: Effects on housing and mental health outcomes. Psychiatric Services. 2015 Apr 15;66(8):806-16.	Wrong study design
21	Hwang SW, Tolomiczenko G, Kouyoumdjian FG, Garner RE. Interventions to improve the health of the homeless: a systematic review. American journal of preventive medicine. 2005 Nov 1;29(4):311-.	Wrong study design
22	Luchenski S, Maguire N, Aldridge RW, Hayward A, Story A, Perri P, Withers J, Clint S, Fitzpatrick S, Hewett N. What works in inclusion health: overview of effective interventions for marginalised and excluded populations. The Lancet. 2018 Jan 20;391(10117):266-80.	Wrong study design
23	Hwang SW, Burns T. Health interventions for people who are homeless. The Lancet. 2014 Oct 25;384(9953):1541-7.	Wrong study design
24	Nelson G, Aubry T, Lafrance A. A review of the literature on the effectiveness of housing and support, assertive community treatment, and intensive case management interventions for persons with mental illness who have been homeless. American Journal of Orthopsychiatry. 2007	Wrong study design

	Jul;77(3):350-61.	
25	Vanderplasschen W, Wolf J, Rapp RC, Broekaert E. Effectiveness of different models of case management for substance-abusing populations. Journal of psychoactive drugs. 2007 Mar 1;39(1):81-95.	Wrong study design
26	Coldwell CM, Bender WS. The effectiveness of assertive community treatment for homeless populations with severe mental illness: a meta-analysis. American Journal of Psychiatry. 2007 Mar;164(3):393-9.	Wrong study design
27	Kasprow WJ, Rosenheck RA. Outcomes of critical time intervention case management of homeless veterans after psychiatric hospitalization. Psychiatric Services. 2007 Jul;58(7):929-35.	Wrong study design
28	Clark C, Guenther CC, Mitchell JN. Case management models in permanent supported housing programs for people with complex behavioral issues who are homeless. Journal of dual diagnosis. 2016 Apr 2;12(2):185-92.	Wrong study design
29	Calsyn RJ, Morse GA, Klinkenberg WD, Trusty ML, Allen G. The impact of assertive community treatment on the social relationships of people who are homeless and mentally ill. Community Mental Health Journal. 1998 Dec 1;34(6):579-93.	Wrong study design
30	Hoell A, Franz M, Salize HJ. Die gesundheitliche Versorgung von wohnungslosen Menschen mit psychischen Problemen. Die Psychiatrie. 2017 Apr;14(02):75-85.	Wrong study design
31	Tomita A, Herman DB. The role of a critical time intervention on the experience of continuity of care among persons with severe mental illness following hospital discharge. The Journal of nervous and mental disease. 2015 Jan;203(1):65.	Irrelevant outcomes
32	Baumgartner JN, Herman DB. Community integration of formerly homeless men and women with severe mental illness after hospital discharge. Psychiatric Services. 2012 May;63(5):435-7.	Irrelevant outcomes
33	Dixon L, Stewart B, Krauss N, Robbins J, Hackman A, Lehman A. The Participation of Families of Homeless Persons with Severe Mental Illness in an Outreach Intervention. Community Mental Health Journal. 1998 Jun 1;34(3):251-9.	Irrelevant outcomes
34	Tomita A, Lukens EP, Herman DB. Mediation analysis of critical time intervention for persons living with serious mental illnesses: Assessing the role of family relations in reducing psychiatric rehospitalization. Psychiatric rehabilitation journal. 2014 Mar;37(1):4.	Irrelevant outcomes
35	Health Quality Ontario. Interventions to improve access to primary care for people who are homeless: A systematic review. Ontario health technology assessment series. 2016;16(9):1.	Irrelevant outcomes
36	Price SK, Gray LA, Thacker LR. Enhanced engagement: an intervention pilot for mental health promotion among low-income women in a community home visiting program. Best Practices in Mental Health. 2015 Mar 1;11(1):69-82.	Wrong intervention
37	Zerger S, Bacon S, Corneau S, Skosireva A, McKenzie K, Gapka S, O'Campo P, Sarang A, Stergiopoulos V. Differential experiences of discrimination among ethnoracially diverse persons experiencing mental illness and homelessness. BMC psychiatry. 2014 Dec;14(1):353.	Wrong intervention
38	Ball SA, Cobb-Richardson P, Connolly AJ, Bujosa CT, O'Neall TW. Substance abuse and personality disorders in homeless drop-in center clients: symptom severity and psychotherapy retention in a randomized clinical trial. Comprehensive psychiatry. 2005 Sep 1;46(5):371-9.	Wrong intervention
39	Nuttbrock LH, Ng-Mak DS, Rahav M, Rivera JJ. Pre-and post-admission attrition of homeless, mentally ill chemical abusers referred to residential treatment programs. Addiction. 1997 Oct;92(10):1305-16.	Wrong intervention
40	Drake RE, Yovetich NA, Bebout RR, Harris M, McHugo GJ. Integrated treatment for dually diagnosed homeless adults. The Journal of Nervous and Mental Disease. 1997 May 1;185(5):298-	Wrong intervention

	305.	
41	Rahav M, Rivera JJ, Nuttbrock L, Ng-Mak D, Sturz EL, Link BG, Struening EL, Pepper B, Gross B. Characteristics and treatment of homeless, mentally ill, chemical-abusing men. Journal of Psychoactive Drugs. 1995 Jan 1;27(1):93-103.	Wrong intervention
42	Dore G, Sinclair B, Murray R. Treatment resistant and resistant to treatment? Evaluation of 40 alcohol dependent patients admitted for involuntary treatment. Alcohol and Alcoholism. 2015 Sep 10;51(3):291-5.	Wrong intervention
43	Broner N, Lang M, Behler SA. The effect of homelessness, housing type, functioning, and community reintegration supports on mental health court completion and recidivism. Journal of dual diagnosis. 2009 Nov 24;5(3-4):323-56.	Wrong intervention
44	Alimohamed-Janmohamed S, Charvat M, Gheytanchi A, Beutler LE, Breckenridge J. Point of entry and functional impairment as predictors of treatment amount and cost for patients with mental illness and substance abuse disorders in Santa Barbara County Mental Health Services. Psychological Services. 2010 Feb;7(1):44.	Wrong intervention
45	Schumacher, J. E., Milby, J. B., Wallace, D., Meehan, D.-C., Kertesz, S., Vuchinich, R., . . . Usdan, S. (2007). Meta-analysis of day treatment and contingency-management dismantling research: Birmingham Homeless Cocaine Studies (1990-2006). Journal of Consulting and Clinical Psychology, 75(5), 823-828. http://dx.doi.org/10.1037/0022-006X.75.5.823	Wrong intervention
46	Zhang H, Neelarambam K, Schwenke TJ, Rhodes MN, Pittman DM, Kaslow NJ. Mediators of a culturally-sensitive intervention for suicidal African American women. Journal of clinical psychology in medical settings. 2013 Dec 1;20(4):401-14.	Wrong intervention
47	Noether CD, Finkelstein N, VanDeMark NR, Savage A, Reed BG, Moses DJ. Design strengths and issues of SAMHSA's women, co-occurring disorders, and violence study. Psychiatric services. 2005 Oct;56(10):1233-6.	Wrong intervention
48	McHugo GJ, Bebout RR, Harris M, Cleghorn S, Herring G, Xie H, Becker D, Drake RE. A randomized controlled trial of integrated versus parallel housing services for homeless adults with severe mental illness. Schizophrenia bulletin. 2004 Jan 1;30(4):969-82.	Wrong intervention
49	Somers JM, Moniruzzaman A, Patterson M, Currie L, Rezansoff SN, Palepu A, Fryer K. A randomized trial examining housing first in congregate and scattered site formats. PloS one. 2017 Jan 11;12(1):e0168745.	Wrong intervention
50	Stergiopoulos V, Gozdzik A, Misir V, Skosireva A, Sarang A, Connelly J, Whisler A, McKenzie K. The effectiveness of a Housing First adaptation for ethnic minority groups: findings of a pragmatic randomized controlled trial. BMC public health. 2016 Dec;16(1):1110.	Wrong intervention
51	Chung TE, Gozdzik A, Palma Lazgare LI, To MJ, Aubry T, Frankish J, Hwang SW, Stergiopoulos V. Housing First for older homeless adults with mental illness: a subgroup analysis of the At Home/Chez Soi randomized controlled trial. International journal of geriatric psychiatry. 2018 Jan;33(1):85-95.	Wrong intervention
52	Adair CE, Streiner DL, Barnhart R, Kopp B, Veldhuizen S, Patterson M, Aubry T, Lavoie J, Sareen J, LeBlanc SR, Goering P. Outcome trajectories among homeless individuals with mental disorders in a multisite randomised controlled trial of housing first. The Canadian Journal of Psychiatry. 2017 Jan;62(1):30-9.	Wrong intervention
53	Aquin JP, Roos LE, Distasio J, Katz LY, Bourque J, Bolton JM, Bolton SL, Wong JY, Chateau D, Somers JM, Enns MW. Effect of Housing First on Suicidal Behaviour: A Randomised Controlled Trial of Homeless Adults with Mental Disorders. The Canadian Journal of Psychiatry. 2017 Jul;62(7):473-81.	Wrong intervention

54	Rezansoff SN, Moniruzzaman A, Fazel S, McCandless L, Procyshyn R, Somers JM. Housing first improves adherence to antipsychotic medication among formerly homeless adults with schizophrenia: results of a randomized controlled trial. Schizophrenia bulletin. 2016 Sep 24;43(4):852-61.	Wrong intervention
55	Kozloff N, Adair CE, Lazgare LI, Poremski D, Cheung AH, Sandu R, Stergiopoulos V. " Housing first" for homeless youth with mental illness. Pediatrics. 2016 Oct 1;138(4):e20161514.	Wrong intervention
56	Aubry T, Goering P, Veldhuizen S, Adair CE, Bourque J, Distasio J, Latimer E, Stergiopoulos V, Somers J, Streiner DL, Tsemberis S. A multiple-city RCT of housing first with assertive community treatment for homeless Canadians with serious mental illness. Psychiatric Services. 2015 Dec 1;67(3):275-81.	Wrong intervention
57	Poremski D, Stergiopoulos V, Braithwaite E, Distasio J, Nisenbaum R, Latimer E. Effects of Housing First on employment and income of homeless individuals: Results of a randomized trial. Psychiatric Services. 2016 Feb 16;67(6):603-9.	Wrong intervention
58	Somers JM, Moniruzzaman A, Palepu A. Changes in daily substance use among people experiencing homelessness and mental illness: 24-month outcomes following randomization to Housing First or usual care. Addiction. 2015 Oct;110(10):1605-14.	Wrong intervention
59	Stergiopoulos V, Hwang SW, Gozdzik A, Nisenbaum R, Latimer E, Rabouin D, Adair CE, Bourque J, Connelly J, Frankish J, Katz LY. Effect of scattered-site housing using rent supplements and intensive case management on housing stability among homeless adults with mental illness: a randomized trial. Jama. 2015 Mar 3;313(9):905-15.	Wrong intervention
60	Aubry T, Tsemberis S, Adair CE, Veldhuizen S, Streiner D, Latimer E, Sareen J, Patterson M, McGarvey K, Kopp B, Hume C. One-year outcomes of a randomized controlled trial of housing first with ACT in five Canadian cities. Psychiatric services. 2015 Apr 21;66(5):463-9.	Wrong intervention
61	Veldhuizen S, Adair CE, Methot C, Kopp BC, O'Campo P, Bourque J, Streiner DL, Goering PN. Patterns and predictors of attrition in a trial of a housing intervention for homeless people with mental illness. Social psychiatry and psychiatric epidemiology. 2015 Feb 1;50(2):195-202.	Wrong intervention
62	Somers JM, Patterson ML, Moniruzzaman A, Currie L, Rezansoff SN, Palepu A, Fryer K. Vancouver At Home: pragmatic randomized trials investigating Housing First for homeless and mentally ill adults. Trials. 2013 Dec;14(1):365.	Wrong intervention
63	Palepu A, Patterson ML, Moniruzzaman A, Frankish CJ, Somers J. Housing first improves residential stability in homeless adults with concurrent substance dependence and mental disorders. American Journal of Public Health. 2013 Dec;103(S2):e30-6.	Wrong intervention
64	Patterson M, Moniruzzaman A, Palepu A, Zabkiewicz D, Frankish CJ, Krausz M, Somers JM. Housing First improves subjective quality of life among homeless adults with mental illness: 12-month findings from a randomized controlled trial in Vancouver, British Columbia. Social psychiatry and psychiatric epidemiology. 2013 Aug 1;48(8):1245-59.	Wrong intervention
65	Hwang SW, Stergiopoulos V, O'Campo P, Gozdzik A. Ending homelessness among people with mental illness: the At Home/Chez Soi randomized trial of a Housing First intervention in Toronto. BMC public health. 2012 Dec;12(1):787.	Wrong intervention
66	Sadowski LS, Kee RA, VanderWeele TJ, Buchanan D. Effect of a housing and case management program on emergency department visits and hospitalizations among chronically ill homeless adults: a randomized trial. Jama. 2009 May 6;301(17):1771-8.	Wrong intervention
67	Stefancic A, Tsemberis S. Housing First for long-term shelter dwellers with psychiatric disabilities in a suburban county: A four-year study of housing access and retention. The journal of primary prevention. 2007 Jul 1;28(3-4):265-79.	Wrong intervention
68	Cherner RA, Aubry T, Sylvestre J, Boyd R, Pettey D. Housing first for adults with problematic	Wrong intervention

	substance use. Journal of dual diagnosis. 2017 Jul 3;13(3):219-29.	
69	Young MS, Clark C, Moore K, Barrett B. Comparing two service delivery models for homeless individuals with complex behavioral health needs: Preliminary data from two SAMHSA treatment for homeless studies. Journal of Dual Diagnosis. 2009 Nov 24;5(3-4):287-304.	Wrong intervention
70	Bradford DW, Gaynes BN, Kim MM, Kaufman JS, Weinberger M. Can shelter-based interventions improve treatment engagement in homeless individuals with psychiatric and/or substance misuse disorders?: a randomized controlled trial. Medical Care. 2005 Aug 1:763-8.	Wrong intervention
71	Zlotnick C, Tam T, Zerger S. Common needs but divergent interventions for US homeless and foster care children: Results from a systematic review. Health & Social Care in the Community. 2012 Sep;20(5):449-76.	Wrong intervention
72	Fitzpatrick-Lewis D, Ganann R, Krishnaratne S, Ciliska D, Kouyoumdjian F, Hwang SW. Effectiveness of interventions to improve the health and housing status of homeless people: a rapid systematic review. BMC public health. 2011 Dec;11(1):638.	Wrong intervention
73	Urbanoski K, Veldhuizen S, Krausz M, Schutz C, Somers JM, Kirst M, Fleury MJ, Stergiopoulos V, Patterson M, Strehlau V, Goering P. Effects of comorbid substance use disorders on outcomes in a Housing First intervention for homeless people with mental illness. Addiction. 2018 Jan;113(1):137-45.	Wrong intervention
74	Kirst M, Zerger S, Misir V, Hwang S, Stergiopoulos V. The impact of a Housing First randomized controlled trial on substance use problems among homeless individuals with mental illness. Drug and alcohol dependence. 2015 Jan 1;146:24-9.	Wrong intervention
75	Nuttbrock LA, Rahav M, Rivera JJ, Ng-Mak DS, Link BG. Outcomes of homeless mentally ill chemical abusers in community residences and a therapeutic community. Psychiatric Services. 1998 Jan;49(1):68-76.	Wrong intervention
76	Lapham SC, Hall M, Skipper BJ. Homelessness and substance use among alcohol abusers following participation in Project H&ART. Journal of Addictive Diseases. 1996 Jan 10;14(4):41-55.	Wrong intervention
77	Sosin MR, Bruni M, Reidy M. Paths and impacts in the progressive independence model: a homelessness and substance abuse intervention in Chicago. Journal of Addictive Diseases. 1996 Jan 10;14(4):1-20.	Wrong intervention
78	Pope AR, Conrad KJ, Baxter W, Elbaum P, Lisiecki J, Daghestani A, Hultman C, Lyons J. Case managed residential care for homeless addicted veterans: Evanston/VA. Alcoholism Treatment Quarterly. 1993 Nov 8;10(3-4):155-69.	Wrong intervention
79	Alphs L, Mao L, Rodriguez SC, Hulihan J, Starr HL. Design and rationale of the Paliperidone Palmitate Research in Demonstrating Effectiveness (PRIDE) study: a novel comparative trial of once-monthly paliperidone palmitate versus daily oral antipsychotic treatment for delaying time to treatment failure in persons with schizophrenia. J Clin Psychiatry. 2014 Dec 1;75(12):1388-93.	Wrong publication
80	VALENCIA E, SUSSER E, TORRES J, FELIX A, CONOVER S. Critical Time Intervention for Homeless Mentally Individuals in Transition from Shelter to Community Living. Mentally ill and homeless: Special programs for special needs. 2013 May 13:75.	Wrong publication
81	Valencia E, Susser E, Torres J, Felix A, Conover S. Critical Time Intervention for Homeless Mentally Individuals. In López, Ibor J. J. Proceedings of the X World Congress of Psychiatry, Madrid, Spain, August 23-28, 1996. Seattle, WA: Hogrefe & Huber, 1999. Print.	Wrong publication
82	Mueser KT, Bond GR, Drake RE, Resnick SG. Models of community care for severe mental illness: a review of research on case management. Schizophrenia bulletin. 1998 Jan 1;24(1):37-74.	Wrong publication

83	Meyer PS, Morrissey JP. A comparison of assertive community treatment and intensive case management for patients in rural areas. Psychiatric services. 2007 Jan;58(1):121-7.	Wrong publication
84	Burns BJ, Santos AB. Assertive community treatment: an update of randomized trials. Psychiatric Services. 1995 Jul 1;46(7):669-75.	Wrong publication
85	Prince JD. Critical time intervention reduces psychiatric rehospitalization among formerly homeless individuals with psychotic diagnoses. Evidence-based mental health. 2013 May;16(2):38-.	Wrong publication
86	Goering P, Veldhuizen S, Nelson GB, Stefancic A, Tsemberis S, Adair CE, Distasio J, Aubry T, Stergiopoulos V, Streiner DL. Further validation of the pathways housing first fidelity scale. Psychiatric Services. 2015 Sep 15;67(1):111-4.	Wrong publication
87	Herinckx HA, Kinney RF, Clarke GN, Paulson RI. Assertive community treatment versus usual care in engaging and retaining clients with severe mental illness. Psychiatric services. 1997 Oct.	Wrong population
88	Lam JA, Jekel JF, Thompson KR, Leaf PJ, Hartwell SW, Florio L. Assessing the value of a short-term residential drug treatment program for homeless men. Journal of Addictive Diseases. 1996 Jan 10;14(4):21-39.	Wrong population
89	Gensichen J, Beyer M, Muth C, Gerlach FM, Von Korff M, Ormel J. Case management to improve major depression in primary health care: a systematic review. Psychological medicine. 2006 Jan;36(1):7-14.	Wrong population
90	Zulman DM, Chee CP, Ezeji-Okoye SC, Shaw JG, Holmes TH, Kahn JS, Asch SM. Effect of an intensive outpatient program to augment primary care for high-need Veterans Affairs patients: a randomized clinical trial. JAMA internal medicine. 2017 Feb 1;177(2):166-75.	Wrong population
91	Stergiopoulos V, Gozdzik A, Cohen A, Guimond T, Hwang SW, Kurdyak P, Leszcz M, Wasylenki D. The effect of brief case management on emergency department use of frequent users in mental health: Findings of a randomized controlled trial. PloS one. 2017 Aug 3;12(8):e0182157.	Wrong population
92	Bell JF, Krupski A, Joesch JM, West II, Atkins DC, Court B, Mancuso D, Roy-Byrne P. A randomized controlled trial of intensive care management for disabled Medicaid beneficiaries with high health care costs. Health services research. 2015 Jun;50(3):663-89.	Wrong population
93	Kuerbis AN, Neighbors CJ, Morgenstern J. Depression's moderation of the effectiveness of intensive case management with substance-dependent women on temporary assistance for needy families: Outpatient substance use disorder treatment utilization and outcomes. Journal of studies on alcohol and drugs. 2011 Mar;72(2):297-307.	Wrong population
94	Killaspy H, Bebbington P, Blizard R, Johnson S, Nolan F, Pilling S, King M. The REACT study: randomised evaluation of assertive community treatment in north London. Bmj. 2006 Apr 6;332(7545):815-20.	Wrong population
95	Calsyn RJ, Yonker RD, Lemming MR, Morse GA, Klinkenberg WD. Impact of assertive community treatment and client characteristics on criminal justice outcomes in dual disorder homeless individuals. Criminal behaviour and mental health. 2005 Dec;15(4):236-48.	Wrong population
96	Dixon L, Weiden P, Torres M, Lehman A. Assertive community treatment and medication compliance in the homeless mentally ill. American Journal of Psychiatry. 1997 Sep 1;154(9):1302-4.	Wrong population
97	Morgenstern J, Neighbors CJ, Kuerbis A, Riordan A, Blanchard KA, McVeigh KH, Morgan TJ, McCrady B. Improving 24-month abstinence and employment outcomes for substance-dependent women receiving temporary assistance for needy families with intensive case management. American journal of public health. 2009 Feb;99(2):328-33.	Wrong population
98	Munoz M, Panadero S. Utility of the interventions for homeless people with mental and/or	Could not be retrieved

	substance use disorders. PSICOLOGIA CONDUCTUAL. 2002;10(2):305-28.	
99	Nordentoft M, Jessen-Petersen B. Homelessness, mental disease and intervention programs in the USA. Ugeskrift for laeger. 1992 Mar;154(10):650-1.	Could not be retrieved

#	Cost and cost effectiveness studies	Reason for exclusion
1	Akin BA, Brook J, Lloyd MH, McDonald TP. Effect of a parenting intervention on foster care reentry after reunification among substance-affected families: A quasi-experimental study. Child maltreatment. 2017 Aug;22(3):194-204.	Wrong population
2	Alimohamed-Janmohamed S, Charvat M, Gheytanchi A, Beutler LE, Breckenridge J. Point of entry and functional impairment as predictors of treatment amount and cost for patients with mental illness and substance abuse disorders in Santa Barbara County Mental Health Services. Psychological Services. 2010 Feb;7(1):44.	Irrelevant outcomes
3	Althaus F, Paroz S, Hugli O, Ghali WA, Daeppen JB, Peytremann-Bridevaux I, Bodenmann P. Effectiveness of interventions targeting frequent users of emergency departments: a systematic review. Annals of emergency medicine. 2011 Jul 1;58(1):41-52.	Wrong intervention
4	Barlow J, Davis H, McIntosh E, Jarrett P, Mockford C, Stewart-Brown S. Role of home visiting in improving parenting and health in families at risk of abuse and neglect: results of a multicentre randomised controlled trial and economic evaluation. Archives of disease in childhood. 2007 Mar 1;92(3):229-33.	Wrong intervention
5	Bassuk EL, DeCandia CJ, Tsertsvadze A, Richard MK. The effectiveness of housing interventions and housing and service interventions on ending family homelessness: A systematic review. American Journal of Orthopsychiatry. 2014 Sep;84(5):457.	Wrong intervention
6	Basu A, Kee R, Buchanan D, Sadowski LS. Comparative cost analysis of housing and case management program for chronically ill homeless adults compared to usual care. Health services research. 2012 Feb;47(1pt2):523-43.	Wrong intervention
7	Bearman D, Claydon K, Kincheloe J, Lodise C. Breaking the cycle of dependency: dual diagnosis and AFDC families. Journal of psychoactive drugs. 1997 Dec 1;29(4):359-67.	Wrong intervention
8	Brush BL, Powers EM. Health and service utilization patterns among homeless men in transition: Exploring the need for on-site, shelter-based nursing care. Scholarly inquiry for nursing practice. 2001 Jan 1;15(2):143-54.	Wrong intervention
9	CADTH. Case management for vulnerable or homeless persons: clinical and cost-effectiveness, and guidelines (structured abstract). 2014 Mar; Project Number RA0670-000	Wrong publication type
10	Dickey B, Latimer E, Powers K, Gonzalez O, Goldfinger SM. Housing costs for adults who are mentally ill and formerly homeless. The Journal of Mental Health Administration. 1997 Jun 1;24(3):291-305.	Wrong intervention
11	Dickey B. Review of programs for persons who are homeless and mentally ill. Harvard review of Psychiatry. 2000 Nov 1;8(5):242-50.	Wrong intervention
12	Drummond J, Wiebe N, So S, Schnirner L, Bisanz J, Williamson DL, Mayan M, Templeton L, Fassbender K. Service-integration approaches for families with low income: a Families First Edmonton, community-based, randomized, controlled trial. Trials. 2016 Dec;17(1):343.	Wrong population
13	Evans WN, Sullivan JX, Wallskog M. The impact of homelessness prevention programs on homelessness. Science. 2016 Aug 12;353(6300):694-9.	Wrong intervention

14	Francis E, Hughes P, Schinka J. Alcohol & Drug Abuse: Improving Cost-Effectiveness in a Substance Abuse Treatment Program. Psychiatric Services. 1999 May;50(5):633-5.	Wrong intervention
15	French MT, McCollister KE, Sacks S, McKendrick K, De Leon G. Benefit–cost analysis of a modified therapeutic community for mentally ill chemical abusers. Evaluation and Program Planning. 2002 May 1;25(2):137-48.	Wrong intervention
16	Gilmer TP, Manning WG, Ettner SL. A cost analysis of San Diego County's REACH program for homeless persons. Psychiatric Services. 2009 Apr;60(4):445-50.	Wrong intervention
17	Gilmer, T. P., Stefancic, A., Ettner, S. L., Manning, W. G., & Tsemberis, S. (2010). Effect of full-service partnerships on homelessness, use and costs of mental health services, and quality of life among adults with serious mental illness. Archives of General Psychiatry, 67(6), 645-652.	Wrong intervention
18	Gulcur L, Stefancic A, Shinn M, Tsemberis S, Fischer SN. Housing, hospitalization, and cost outcomes for homeless individuals with psychiatric disabilities participating in continuum of care and housing first programmes. Journal of Community & Applied Social Psychology. 2003 Mar;13(2):171-86.	Wrong intervention
19	Hadley J, Holahan J. Is health care spending higher under Medicaid or private insurance?. INQUIRY: The Journal of Health Care Organization, Provision, and Financing. 2003 Nov;40(4):323-42.	Wrong intervention
20	Holter MC. An intervention to reduce recurrent homelessness among severely mentally ill men: Benefit-cost analysis.	Wrong intervention
21	Holtgrave, D. R., Wolitski, R. J., Pals, S. L., Aidala, A., Kidder, D. P., Vos, D., ... & Bendixen, A. V. (2013). Cost-utility analysis of the housing and health intervention for homeless and unstably housed persons living with HIV. AIDS and Behavior, 17(5), 1626-1631.	Wrong intervention
22	Healthcare Costs Reduced When Patients Have a Place to Live. Hospital Case Management: The Essential Guide to Hospital-Based Care Planning; Atlanta Vol. 25, Iss. 7, (Jul 2017).	Wrong publication type
23	Hwang SW, Weaver J, Aubry T, Hoch JS. Hospital costs and length of stay among homeless patients admitted to medical, surgical, and psychiatric services. Medical care. 2011 Apr 1:350-4.	Wrong intervention
24	Johnson P. Medicaid: Medicaid: provider reimbursement--2005. End of Year Issue Brief. Issue Brief (Health Policy Tracking Service). 2005 Dec:1-1.	Wrong population
25	Kleinman R, Kehn M, Wishon Siegwarth A, Brown J. State strategies for coordinating Medicaid and housing services. Psychiatric rehabilitation journal. 2017 Jun;40(2):225.	Wrong intervention
26	Larimer ME, Malone DK, Garner MD, Atkins DC, Burlingham B, Lonczak HS, Tanzer K, Ginzler J, Clifasefi SL, Hobson WG, Marlatt GA. Health care and public service use and costs before and after provision of housing for chronically homeless persons with severe alcohol problems. Jama. 2009 Apr 1;301(13):1349-57.	Wrong intervention
27	Lenz-Rashid, S. (2017) Supportive housing program for homeless families: Foster care outcomes and best practices.	Wrong intervention
28	Lim S, Gao Q, Stazesky E, Singh TP, Harris TG, Seligson AL. Impact of a New York City supportive housing program on Medicaid expenditure patterns among people with serious mental illness and chronic homelessness. BMC health services research. 2018 Dec;18(1):15.	Wrong intervention
29	Ly A, Latimer E. Housing first impact on costs and associated cost offsets: a review of the literature. The Canadian Journal of Psychiatry. 2015 Nov;60(11):475-87.	Wrong intervention
30	Mangalore R, Knapp M. Cost of schizophrenia in England. The journal of mental health policy and economics. 2007 Mar;10(1):23-41.	Wrong population

31	Manuel JI, Covell NH, Jackson CT, Essock SM. Does assertive community treatment increase medication adherence for people with co-occurring psychotic and substance use disorders?. Journal of the American Psychiatric Nurses Association. 2011 Jan;17(1):51-6.	Irrelevant outcomes
32	Marks SM, Taylor Z, Burrows NR, Qayad MG, Miller B. Hospitalization of homeless persons with tuberculosis in the United States. American Journal of Public Health. 2000 Mar;90(3):435.	Wrong intervention
33	Masson CL, Sorensen JL, Batki SL, Okin R, Delucchi KL, Perlman DC. Medical service use and financial charges among opioid users at a public hospital. Drug and Alcohol Dependence. 2002 Mar 1;66(1):45-50.	Wrong intervention
34	McCormick B, White J. Hospital care and costs for homeless people. Clinical Medicine. 2016 Dec 1;16(6):506-10.	Wrong intervention
35	McLaughlin, T. (2011). Using common themes: Cost-effectiveness of permanent supported housing for people with mental illness. Research on Social Work Practice, 21(4), 404-411.	Wrong intervention
36	McMorrow S, Kenney GM, Anderson N, Clemans-Cope L, Dubay L, Long SK, Wissoker D. Trade-offs between public and private coverage for low-income children have implications for future policy debates. Health Affairs. 2014 Aug 1;33(8):1367-74.	Wrong population
37	Pauley et al. 2016 Pauley T, Gargaro J, Falode A, Beben N, Sikharulidze L, Mekinda B. Evaluation of an integrated cluster care and supportive housing model for unstably housed persons using the shelter system. Professional case management. 2016 Jan 1;21(1):34-42.	Wrong intervention
38	Poulin SR, Maguire M, Metraux S, Culhane DP. Service use and costs for persons experiencing chronic homelessness in Philadelphia: a population-based study. Psychiatric Services. 2010 Nov;61(11):1093-8.	Wrong intervention
39	Hunter, Sarah B., Melody Harvey, Brian Briscombe, and Matthew Cefalu, Evaluation of Housing for Health Permanent Supportive Housing Program. Santa Monica, CA: RAND Corporation, 2017. https://www.rand.org/pubs/research_reports/RR1694.html	Wrong intervention
40	Read S. Health and homelessness-a whole-systems perspective. Housing, care and support. 2008 Jul 1;11(1):7-10.	Wrong intervention
41	Rosenheck R, Lam J, Randolph F. Impact of representative payees on substance use by homeless persons with serious mental illness. Psychiatric Services. 1997 Jun.	Wrong intervention
42	Rosenheck R, Seibyl CL. Homelessness: health service use and related costs. Medical care. 1998 Aug 1:1256-64.	Wrong intervention
43	Sacks S, McKendrick K, Sacks JY, Cleland CM. Modified therapeutic community for co-occurring disorders: single investigator meta analysis. Substance Abuse. 2010 Jul 30;31(3):146-61.	Wrong intervention
44	Schinka JA, Francis E, Hughes P, LaLone L, Flynn C. Comparative outcomes and costs of inpatient care and supportive housing for substance-dependent veterans. Psychiatric Services. 1998 Jul;49(7):946-50.	Wrong intervention
45	Schumacher JE, Mennemeyer ST, Milby JB, Wallace D, Nolan K. Costs and effectiveness of substance abuse treatments for homeless persons. Journal of Mental Health Policy and Economics. 2002 Mar;5(1):33-42.	Wrong intervention
46	Torrey EF. Economic barriers to widespread implementation of model programs for the seriously mentally ill. Psychiatric Services. 1990 May;41(5):526-31.	Wrong intervention
47	De Vet R, van Luijtelaar MJ, Brilleslijper-Kater SN, Vanderplasschen W, Beijersbergen MD, Wolf JR. Effectiveness of case management for homeless persons: a systematic review. American Journal of Public Health. 2013 Oct;103(10):e13-26.	Wrong study design

48	Zur J, Mojtabai R, Li S. The cost savings of expanding Medicaid eligibility to include currently uninsured homeless adults with substance use disorders. The journal of behavioral health services & research. 2014 Apr 1;41(2):110-24.	Wrong intervention
