# Supplementary material for: The effectiveness of case management interventions for the homeless, vulnerably housed and persons with lived experience: A systematic review
Source: PLoS One. 2020 Apr 9;15(4):e0230896. doi: 10.1371/journal.pone.0230896 (PMC7313544; doi:10.1371/journal.pone.0230896)
Supplement: S4 File — (PDF) [file pone.0230896.s004.pdf]

Appendix IV: Characteristics of Included Studies (All)

Appendix 4.1: Characteristics of Included Studies (SCM):

Study ID	Publication related to this study	Study design	Study follow-up	Study population	Study setting	Sample size	Intervention	Comparator	Outcomes of interest	Findings
Conrad et al. 1998 [44]	N/A	Randomized control trial	24 months	Homeless addicted male veterans	Illinois<br><br>United States	Total sample n=358 Intervention n=178 Control n=180	**Intervention**<br>3-6 months of stay at hospital facility (residential care) plus case management. The ratio of residents to case managers was 10:1 in the residency phase and approximately 25:1 in the community follow-up phase. Relapse prevention skills training, consisting of assertive drink and drug refusal, coping with relapse, social networking, and anger management, was an essential component of CMRC treatment. Self-help groups such as Narcotics Anonymous (NA) and Alcoholics Anonymous (AA), both on-site and community-based, were emphasized for emotional support and to enhance coping behavior while achieving abstinence	**Control:**<br>21 days in customary care: During the hospital stay in customary care, patients received substance abuse and abstinence education and individual and group therapy. Patients were seen by a social worker for assessment, psychotherapy, and discharge planning.	Housing stability, Substance use, Employment	Even though the experimental group reported fewer number of nights homeless than the control group at 12 months, this effect was reversed at 24 months and the statistical significance diminished by time. No significant differences between groups on ASI's psychiatric symptoms subscore. Both groups improved on the alcohol and the drug use composite scores with those in the intervention group reporting better improvements over 2 years. The improvements, however, tended to decrease with time and the statistical significance diminished as well. Both groups reported improvements in employment outcomes with a statistically significant between-group difference favouring the intervention group over the entire study period.
Graham-Jones et al. 2004 [45]	Reilly et al. 2004[46] is a companion study (Same intervention, different sample)	Quasi-experimental 3-armed controlled trial	3 years	Homeless patients registering on a temporary basis at Prince Park Health Centre	Liverpool<br><br>United Kingdom	Total sample n=117 Health centre advocacy n=22 Outreach advocacy n=53 control n=42	**Health centre advocacy group:**<br>During 'intervention' months, receptionists registering temporary patients from homeless families at the health centre put these patients in touch with the health advocate (family health worker, FHW) before or soon after their first consultation with a GP.<br><br>**Outreach advocacy group:**<br>Outreach visits by the FHW to hostels and bed and breakfast hotels during intervention months allowed newly arrived homeless individuals and families to be proactively registered as temporary patients. Health advocacy work could then be initiated early in their	**Control group (usual care):**<br>During 'control' months, new temporary resident patients registered themselves at the health centre and accessed usual care (including appointments/home visits with a GP or visits to the practice nurse).	Housing stability, Quality of life	Even though a higher percentage of participants in the outreach advocacy group were rehoused or achieved a positive housing outcome than the health centre advocacy group or the control group, those differences were not statistically significant. When looking at the Nottingham Health profile, those in the health centre group reported better improvements than the control group on the social isolation dimension only, whereas those in the outreach group had better improvement on the emotional distress and sleep subscores. When looking at the Life fulfilment scale (LFS), even though both intervention groups showed

							stay in the area.			improvements on different subscales compared to the control group, only the outreach advocacy group improved significantly on the aggregated fulfilments scales. Finally, when looking at the Delighted-Terrible faces scale; no significant differences were found between groups. A health service utilization analysis reported no significant difference between groups on number of days hospitalized.
Hurlburt et al. 1996 [47]	N/A	4 arm randomized control trial	24 months	Homeless or at high risk of becoming homeless individuals diagnosed with severe and persistent mental illness (Schizophrenia, major depression, or bipolar disorder)	San Diego CA<br><br>United States	Total sample n=361<br>Comprehensive CM with vouchers n=90-91<br>Comprehensive CM without vouchers n=90-91<br>Traditional CM with vouchers n=90-91<br>Traditional CM without vouchers n=90-91	Section 8 housing certificates from the Department of Housing and Urban Development (HUD) to local housing authorities in San Diego. These certificates are designed to make it possible for low income individuals to choose and obtain independent housing in the community.<br><br>Comprehensive case management was provided by a private mental health service under contract with the county and differed from the traditional condition in several respects- comprehensive case managers had smaller maximum caseloads (22 vs.40); were available to clients 24 hours a day, seven days per week; and had higher salaries. They took a formal team approach to working with clients, attempted to establish housing support groups for participants in housing, and tried to work with clients on finding employment.	Traditional Case management with or without HUD-VASH Section 8 housing vouchers	Housing stability	No significant improvements were found on housing outcomes for participants randomized to the comprehensive case management program compared to those randomized to traditional case management
Lapham et al. 1995 [48]	N/A	4 arm randomized control trial	10 months	Homeless alcohol abusers	New Mexico<br><br>United States	Total sample n=469<br>Group 1 n=161<br>Group 2 n=164<br>Group 3 n=92<br>Group 4 n=52	**Group 1**, the high intensity group, received case management and substance abuse counselling services, along with four months of housing in four-plex apartment buildings staffed by residence managers who provided peer support<br><br>**Group 2**, medium intensity group, received four months of housing in similar apartments with support services from peer	**Group 3**, low intensity group, received four months of apartment- or motel-based housing and no additional services.<br><br>About halfway through the 16-month intervention phase, Group 3 housing services were discontinued due to safety concerns for staff and clients. Individuals randomized to the new low intensity nonhoused group (**designated Group 4**) received referrals and bus fare to local and statewide alcohol	Housing stability, Substance use, Employment	No statistically significant between-group differences were found for number of days in stable housing, number of days of alcohol use, and number of days participants were employed

							residence managers. Clients in Group 2 were expected to seek treatment for their alcohol and drug abuse on their own initiatives, from services normally available in the community.	treatment agencies and were paid to provide health services utilization data at twice weekly check-ins.		
Nyamathi et al. 2001 [49]	N/A	3-arm randomized control trial	6 months	Homeless women and their intimate partners	Los Angeles, California<br><br>United States	Total sample n=948<br>Peer-mentored group n=258<br>Nurse case managed program n=360<br>Standard care program n=330	**Nurse Case-managed program**<br>Over the six weekly sessions the couple received, in group format with one or two other couples, information on HIV/AIDS, risk behaviors, and risk-reducing and health-protecting behaviors. Entry into needed agencies, such as outpatient services, clinics, and social services, was facilitated. As well as ongoing assistance in obtaining needed health care services.<br><br>**Peer intervention**<br>Women and their intimate partners assigned to the peer-mentored program received the same intervention as those in the nurse case-managed program, except that the role of the nurse was assumed by a female peer mentor who matched the participants' ethnicity. Now sober and living in stable home environments, peer mentors were trained extensively by the research team to administer the peer-mentored program and questionnaires, as well as to facilitate referrals to health and social services.	**Standard Care**<br>Participants were administered the instrument packet by the research staff and received a standard traditional 15-min HIV antibody pretest as well as posttest counseling by the research nurses or outreach workers. HIV pretest counseling included an assessment and discussion of drug and sexual behaviors that place one at risk for HIV/AIDS and an explanation of the meaning of negative and positive HIV antibody test results. HIV posttest counseling reinforced this information, provided the result of the HIV antibody test, and reinforced the meaning of either the negative or positive test result.	Mental health, Quality of life, Substance use	Those in the NCM group had relatively low psychological wellbeing scores than the control group. Depression symptoms lessened in the control group whereas it remained the same for the NCM group. Furthermore, when controlling for baseline variables, NCM participants were more likely to have high levels of hostility compared to the control group. No statistically significant differences were found for quality of life or substance use outcomes
Nyamathi et al. 2016 [50]	N/A	Randomized control trial	12 months	Homeless men recently released from California jails and prisons	California<br><br>United States	Total sample n=600<br>PC-NCM n=195<br>PC n=196<br>Usual care n=209	PC-NCM: A peer coach spent 45 min on a weekly basis with each assigned participant. The main focus of each session included building effective coping skills, personal assertiveness, self-management, therapeutic nonviolent communication (NVC), and self-esteem building. Over an 8-week period, this program-specific nurse provided culturally competent NCM for about 20 min each week per person for the PC-NCM group, which focused on health promotion, completion of drug	The usual care subjects received all recovery and rehabilitation services available at the RDT site, including substance abuse services, assistance with independent living skills, job skills assistance, literacy, various counseling services, and discharge planning. The only differences were the absence of the two configurations of peer coaching and/or nurse-led case management	Housing stability, substance use, employment	Even though participants in all three groups reported better substance use outcome, no statistically significant between group differences were observed in this outcome, nor was it observed in housing stability or employment outcomes

							treatment, vaccination compliance, and reduction of risky drug and sexual behaviors.<br><br>Intermediate peer coaching Participants received weekly peer coaching interaction similar to the PC component of the PC-NCM program, but without the NCM component. Although without regular NCM, a second nurse provided a brief, 20-min education session on hepatitis prevention and HIV risk reduction.			
Sosin et al. 1995 [51]	N/A	3-arm randomized control trial	12 months	Graduates of a short-term inpatient substance use programs who lacked housing	Chicago, Ill<br><br>United States	Total sample size n= 419 CM only n=96 CM with Supported housing n=136 control n=187	The **case management only intervention** provided the progressive independence case management services under a scheme in which workers also helped clients find housing in the community. The **housing intervention** provided the case management model along with supported housing in one of three blocks of twenty apartments, found in recently renovated buildings serving those with low incomes. Both interventions were meant to last for up to eight months, although less intensive case management services could (but rarely did) last longer. Those who suffered two relapses or repeatedly violated program rules could not remain in the housing. They could continue case management as long as they agreed to a new contract that would guard against further relapses.	The clients placed in the **control condition** were referred by the relevant short-term program staff to an outpatient or inpatient substance abuse agency, to welfare offices (as needed), and to an address of some kind. In the current paper, we ask whether, and by what mechanisms, each of the two treatment interventions reduced substance abuse<br>and homelessness beyond the progress achieved by individuals placed in the control condition.	Housing stability, Substance use	The case management only condition significantly increased the average number of days of residential stability and reduced the reported average days of alcohol and drug consumption
Towe et al. 2019[52]	N/A	Randomized control trial	12 months	Homeless single adults living in HIV emergency shelters	New York, NY<br><br>United States	Total sample size n=236 EHPA n=119 Usual care n=117	Enhanced Housing Placement Assistance (EPHA) is a rapid-rehousing program where participants were immediately assigned a case manager who worked to identify available and affordable housing for participants as quickly as possible, provided rent and move-in assistance, and delivered intensive housing stabilization services up to one year post-enrollment	Usual care participants were immediately referred to an organization that assists with finding housing for people living with HIV/AIDS and offering housing stabilization services as needed within 3 month post enrollment	Housing stability	At 12 months, a higher percentage of participants receiving a rapid rehousing case management were placed in stable housing compared to usual care.
Upshur et	N/A	Randomized	6	Homeless women	Not	Total sample	**Project RENEWAL**	**Usual care**:	Housing	Even though no statistically

al. 2015 [53]		control trial	months	with alcohol use problems	specified<br><br>United States	n=82 Intervention n=42 Control n=40	**intervention**: Intervention patients received the guideline-based Primary Care Provider brief intervention for problem alcohol use, and referral to the CM for ongoing follow-up visits for 6 months. 1) providing evidence-based training and supports to the medical leadership and randomized intervention PCPs; 2) modifying the electronic medical record (EMR) to provide alcohol screening results and alcohol-specific notes for PCP and care manager (CM) visits; and 3) training a CM specifically designated to provide intervention participants with alcohol education materials, ongoing self-management support, linkage to formal addiction treatment services and self-help groups, and wellness counseling and goal setting.	Usual care patients did not receive referrals to, or outreach from, the study-trained CM, and their PCPs were not provided any alcohol intervention training or patient materials. They delivered usual care for medical conditions, including any behavioral health or drug or alcohol use problems.	stability, Mental health, Substance use	significant difference between groups was found on housing outcomes, women in the intervention group had double the time in their own apartment than those in the intervention group. Conversely, women in the usual care group had significantly fewer nights in shelter than those in the intervention group. No statistically significant differences were found on mental health status. However, a significantly lower percentage of participants met depression criteria at follow-up. No significant between-group differences were found on alcohol consumption rates, percentage of completely abstained participants, or negative consequences of alcohol use. A significantly higher number of days of drug use were reported by intervention participants compared to the usual care group.
Weinreb et al. 2016 [54]	N/A	Cluster randomized control trial	6 months	Homeless mothers who screened positive for major depressive disorder	Queens and Bronx, New York<br><br>United States	Total sample n=67 Intervention n=42 Control n=25	**Intervention**: Integrated Care Model for Homeless Mothers (ICMHM): provided an appointment with the primary care physician (PCP) and with the care manager to initiate depression treatment following the intervention model and to address any other health care needs	**Usual Care**: provided appointments with the PCP who initiated treatment as usual, which could include antidepressant medication and recommendation for psychotherapy outside the clinic. Women in the usual-care group received general case management services that were available to all families receiving health services at the clinic. These services included, for example, assistance with obtaining public benefits, linking with community resources for family activities, outside mental health or substance use services, and meeting children's educational needs.	Housing stability, Mental health, Employment	Both groups reported similar modest housing improvements, with more than half of participants still living in the shelter. No significant between group difference was found for depression symptoms. Even though a higher percentage of participants in the intervention group reported acquiring employment, no significant difference was found between groups.

Appendix 4.2: Characteristics of Included studies (ICM):

Study ID	Publication related to this study	Study design	Study follow-up	Study population	Study setting	Sample size	Intervention	Comparator	Outcomes of interest	Findings
Braucht et al. 1995 [64]	N/A	Randomized control trial	10 months	Homeless individuals 18 years or older	Denver, Colorado	Total sample n=323 Intervention	Intensive Case Management (ICM) with access to services available at Arapahoe House (detoxification facility). ICM	Access to the full range of services offered by Arapahoe House. Services include a	Housing stability, Mental health, Quality of	ICM participants reported significantly less drug use during the last 30 days than

				with alcohol or other substance abuse problems	United States	n=163 control n=160	included proactive outreach, client identification and assessment, development of an individually-tailored and comprehensive service plan for each client, establishment of linkages between service systems and clients such that services were matched to client needs, continuity of service utilization monitoring, and assertive advocacy with community agencies on behalf of clients. Case managers worked in pairs (dyads) with a caseload averaging 15 clients per dyad (and never exceeding 17 clients per dyad) and with a consequent high intensity of client contact.	comprehensive array of substance abuse treatment and rehabilitation services, including detoxification, residential and outpatient services, substance abuse counseling, literacy and vocational assessment, and job training and placement.	life, Substance use, Employment	the control group, whereas those randomized to the control group reported better improvements in their general life satisfaction than the ICM group. ICM had little and non-significant effect on housing stability, mental health, and employment outcomes.
Burnam et al. 1995 [65]	N/A	3-arm Randomized control trial	9 months	Homeless adults with both a serious mental illness and substance dependence	The Westside area, Los Angeles county<br><br>United States	Total sample size n=276 Experimental arm n=144 Experimental arm2 n=67 Control n=65	**Experimental arm 1**: A social model residential program providing integrated mental health and substance abuse treatment<br><br>**Experimental arm 2:** A community-based nonresidential program using (1) curriculum-based groups focused on substance abuse and mental health education and rehabilitation; (2) 12-step programs including participation in community-based AA or NA meetings (3)discussion of issues of importance to the clients (4) individual counseling and case-management (5) psychiatric consultation and ongoing medications management (6) general community activities	Participants randomized to the control group did not receive interventions but were allowed free access to community services	Housing stability, mental health, substance use	There was no significant difference in the percentage of time spent on streets between residential and non-residential groups, or between both treatment groups and control over a 9-month follow up. Furthermore, there were no significant differences in mental health symptoms (depression and anxiety, psychotic symptoms, anger and hostility, mania, self-esteem) between residential and non-residential groups or between both treatment groups and control at 9 months. There were no significant difference in substance use in past 30 days (days used alcohol, level alcohol use, days used drugs, severity of drug use) between all groups.
Cauce et al. 1994[66]	N/A	Randomized control trial	3 months	Homeless adolescents 13-21 years old	Seattle, Washington<br><br>United States	Total sample size n=115 Intervention n=55 control n=60	Intensive case management: Development of treatment plans, linkage to appropriate services, monitor and track clients, advocating for basic entitlements, 24 hour crisis service. Maximum caseload of 12	Those randomized to the control group received regular case management with caseload 18-30	Mental health, quality of life, substance use	No statistically significant differences between groups were found on mental health and substance use outcomes. Life Domain Scale (LDS) scores indicated a significantly increasing satisfaction rate for youths in Project Passage at 3 months compared the control group.
Clark et al. 2003[67]	N/A	Quasi experimental trial	12 months	Homeless adults with mental illness	Pinellas county, Florida<br><br>United States	The total sample size n=152 Comprehensive housing program n=83	**Comprehensive housing programs:** Developed a program specifically to prevent and reduce homelessness in this population. The program features guaranteed access to housing and housing support services, case	**Case management only:** developed a homeless outreach and support team (HOST) to provide short-term case management services for homeless individuals with	Housing stability, mental health	High impairment participants in the ICM only group showed less improvement in stable housing and less reduction in functional

						Case management n=69	management, and priority access to everything from medication management to vocational services. Project Return in Tampa, Florida, also provides comprehensive housing services to homeless persons with severe mental illness, including guaranteed access to housing, housing support services, and case management.	severe mental illness. The activities of this blended case management program (11) include active outreach and engagement, some on site counseling, medication and medication management, assistance with obtaining housing, and linkages to other psychosocial services.		homelessness than did high-impairment participants in the comprehensive housing program group. There were no significant differences between treatment groups for low and medium impairment.
Cox et al. 1998 [68]	Cox et al. 1993[58]	Randomized control trial	2 years	Homeless chronic inebriate clients	Seattle, Washington<br><br>United States	Total sample n=289<br>ICM n=150<br>usual care n=148	Intensive Case management: long-term, open-ended, outreach-oriented service focused primarily on system advocacy and linkage activities. Retention was regarded as more important than compliance, so provision of the service was not conditional on client behaviour and there was no requirement that clients maintain sobriety in order to continue in the program. Caseloads averaged 15 clients per case manager	Those randomized to the control group received standard treatment	Housing stability, Substance use, Employment, Income	Participants in the ICM group reported significantly fewer days homeless, more days stably housed and less alcohol use than those in the usual care group. No statistically significant between-group differences were found on income and employment outcomes with an exception of a significant group effect favouring the ICM group on monthly public income assistance.
Felton et al. 1995 [70]	N/A	3-arm quasi experimental trial	18-months follow-up	Homeless individuals, long-term psychiatric inpatients, and heavy users of emergency services with serious and persistent mental illness	Bronx, New York city<br><br>United States	Total sample size n=104<br>ICM-peer n=36<br>ICM-para n=36<br>ICM-only n=32	**ICM-peer:** Three consumer peer specialists were added to one unit, received 8 week of case management training before assignment and additional training in peer counselling and self-help.<br><br>**ICM-para:** 3 Bronx residents with no previous experience as mental mental health consumers or provided were added to a second unit as para-professional and received 8 weeks of case management training before assignment	ICM-Only: Key program features include small caseload(roughly 10 clients/worker), 24hr availability of staff, a rehabilitation orientation, and assertive outreach and advocacy	Quality of life , Mental health	When comparing the ICM-para group to the ICM only group, no significant between-group differences were detected on any outcome measure. However, high-order group by-tim interactions were significant for satisfaction with finances and social relations.<br><br>When comparing the ICM-peer group to the other two groups, linear group-by-time interactions were significant for satisfaction with living situations, finances and fewer life problems subscores.
Grace et al. 2014[71]	N/A	Non-randomized control trial	24 months	Unemployed and homeless young people	Victoria<br><br>Australia	Total sample n=422<br>Intervention n=235<br>control n=187	Intensive client-centered case management, involving direct provision of a range of services as well as the brokering of additional services, all through a single point of contact—the YP case manager. The intervention was not standardised in terms of duration and intensity. The defining feature was that J group participants remained eligible for joined up services, and were entitled to re-engage with those services at any time during the service delivery phase of the trial. At the end of the service delivery phase of the trial, J	S group remained eligible for standard services available in the community, delivered by a range of government and community-based organisations including housing, employment, counselling, and health services, but without the joining up and single point of contact that were characteristics of the YP4 joined up services that were available to J group. The	Housing stability, Income	Participants in both groups reported improvements in housing stability and income outcomes. However, no statistically significant between-group differences were found on any outcome variable

							group reverted to being eligible for standard services.	mode of service delivery was the key difference between the two groups. Standard service delivery involved clients in complex circumstances receiving multiple and potentially uncoordinated services from different providers.		
Korr et al. 1996 [72]	N/A	Randomized control trial	6 months	homeless mentally ill adults aged 18 or older	Chicago, Ill<br><br>United States	Total sample n=114<br>Intervention n=48<br>control n=47<br>At risk n=19	Bridge services that provide assertive outreach and service coordination through staff who work entirely on the street and in the homes of clients to link the client to entitlements such as the Supplementary Security Income and to mental health treatment services, especially medication. They also assist in teaching living skills, linking to rehabilitative services including supported employment. In most cases, the agency also serves as a representative payee to receive the client's disability check	Control: whatever community services were available at the time of discharge	Housing stability, Hospitalization	Participants receiving the intervention were significantly more likely to be housed than those in the control group. However, no statistically significant difference was found between groups on number of days in the hospital.
Malte et al. 2017[73]	N/A	Randomized control trial	12 Months	Participants were homeless veterans enrolled in addictions treatment, predominantly male and unmarried	Seattle TX<br><br>United States	Total sample size: 181<br>ICM n=91,<br>HSG control n=90	ICM: Caseload was 20. Case management provided (a) support in obtaining/maintaining housing through education about resources, coordination with VA and community housing program providers, assistance in establishing housing program eligibility, and problem solving around threats to housing stability; (b) support for SUD and related issues that affect housing status through treatment engagement/reengagement, referrals for needed services (e.g., psychiatric, medical, vocational), and addressing substance use issues proactively; and (c) promotion of residential stability through life skills training	HSG (control): drop-in housing support group held weekly in the Addiction Treatment Center. The group focused on gaining support from fellow study participants and learning from those who successfully obtained housing. Group facilitators provided education about housing resources and assistance with housing-related issues.	Housing stability, mental health, substance use, hospitalization	There were no statistically significant differences between groups on housing stability or mental health outcomes. Even though participants in the intervention group showed greater improvements in the ASI alcohol composite score compared to those in the control group, no significant between-group difference was found on the proportion of participants who are abstinent from alcohol or drugs. Conversely, participants in the control group reported a significant decrease in emergency department visits compared to the intervention group.
Marshall et al. 1995 [74]	N/A	Randomized control trial	14 months	Homeless individuals with severe and persistent psychiatric disorders	Oxford<br><br>United Kingdom	Total sample n=80<br>Intervention n=40<br>Control n= 40	Case-managers chose how much time to offer each subject. Each client was offered an assessment of need from a case-manager, a discussion of the findings of this assessment with the subject's career, intervention from the case manager to meet needs that were identified, monitoring of the subject's progress by the case-manager and further assistance should needs arise. Case-managers were free to choose how	Those randomized to the control group continued to receive any assistance that they had been receiving before the study	Housing stability, mental health, quality of life, hospitalization, Employment	Even though participants receiving ICM reported more days in better accommodation, less days in worse accommodation, and more days in any kind of employment than the control group, this difference was not statistically significant on any of the outcome variables. Furthermore, after adjusting

							far they would personally assist the subject with transport, counselling, organization of activity programmes, assistance with completion of forms, crisis intervention, help with finding accommodation, assistance with benefits, finding work or places on training courses, and help with obtaining furnishings and domestic appliances.			for hospital days during the baseline period, no significant between group difference was found on number of days in hospital. There were no significant improvements in quality of life or mental health outcomes.
Orwin et al. 1994 [75]	N/A	3- arm randomized control trial	24 months	Homeless persons with alcohol or other drug use problems	Minneapolis<br><br>United States	Total sample n=260<br>ICM n=82<br>Intermediate CM n=117<br>control n=61	**Intensive Case Management:** The intensive case managers were to focus more on outreach and field work, maintaining closer and more frequent contact with clients and in a variety of settings. The intensive group was designed on a modified team model that periodically redistributed clients among the team so that team members became familiar with each other's clients.	**Intermediate Case Management:** Intermediate case managers were expected to be office based, although their goals also included outreach. Managers were not expected to develop close relationships with the clients. The majority of their time was spent on practical issues, such as assisting with entitlement procurement and establishing representative payee arrangements.<br><br>**Control group (Episodic or Usual care):** This group received only episodic case management services- the services normally available through the county.	Housing stability, mental health, substance sue, employment	Data analysis procedures were not described properly. Participants in the control group reported significantly better improvements in housing stability, mental health, substance use and employment outcomes than those in the intervention group.
Rosenblum et al. 2002 [76]	N/A	Quasi-experimental trial	4 months	Homeless substance users	New York city<br><br>United States	Total sample n=250<br>[Description not provided]	Experimental subjects are seen by a social worker at the time that they are recruited for the study. The social worker provides intensive case management (ICM), which includes a comprehensive needs assessment, multiple sessions to increase probability of appropriate and completed referrals, and incentives (such as phone cards, public transportation tokens, waist wallets, grooming and sanitary supplies) for service engagement.	Control subjects could choose to refer themselves to the social worker. They are not given incentives for repeated sessions	Housing stability, substance use, hospitalization, income	All participants reported improvements in housing and substance use outcomes with no significant between-group difference found. However, participants in the ICM group reported fewer hospital emergency room visits and higher receipt of public income assistance than those in the control group.
Shern et al. 2000[77]	N/A	Randomized control trial	24 months	Street-dwelling individuals with psychiatric disabilities	Manhattan, New York<br><br>United States	Total sample n=168<br>Intervention n=91<br>control n=77	Intervention participants had access to the Choices program which included: 1. Outreach and engagement designed to foster the development of rudimentary relationships between Choices staff and homeless individuals 2. Invitation to attend and join the Choices center, where resources (showers, food) were available for experimental study participants 1am-7pm daily. Participation in structured group activities was not required, but assistance was available to anyone requesting help in obtaining health,	Control participants received information about "standard treatment" - that is the existing array of homelessness and specialty mental health services in New York City.	Housing stability, mental health, quality of life	Participants receiving the intervention reported better improvements in housing stability, mental health, and quality of life outcomes than those in the control group.

							mental health, dental and social services and in developing and implementing individual rehabilitation plans. Additionally, the center provided an opportunity for members to meet new friends and socialize. 3. Respite housing in 10 bed, informal church-based shelters or in blocks of YMCA rooms rented by the program and overseen by program staff. 4. In-community and on-site rehabilitation services to assist individuals in finding and maintaining community-based housing. The Choices program structure was similar to an ICM program with a 13:1 client to case manager ratio.			
Stahler et al. 1995 [78]	N/A	Randomized control trial	6 months	Adult males experiencing homelessness with alcohol and/or drug problems and stable mental health	Philadelphia United States	Total sample size n=722 Group 1 n=220 Group 2 n=200 Group 3 n=302	**Group 1**: Integrative Comprehensive Residential Services: A 6-month treatment program with a variety of services on site, such as individual counselling, group therapy, lectures, life skills preparation, job search skills training, and vocational and educational training **Group 2:** On-Site Shelter-Based Intensive Case Management: 4 to 9 months of ICM, case workers referred patients to a community network of services, caseloads were approximately 15 clients per case manager	**Group 3:** Usual Care Shelter Services with Case Management: City-staffed case managers with caseloads of approximately 50-75 per manager. The case managers primarily linked with ancillary supportive services and aided them in finding stable housing	Housing stability, mental health, substance use, employment	Even though both groups reported improvements in housing stability, mental health, substance use and employment outcomes, no statistically significant between-group differences were found on any of these outcomes measured. When subgroup analyses were made based on treatment attrition, results varied.
Shumway et al. 2008 [79]	N/A	Randomized control trial	24 months	Homeless or vulnerably housed frequent emergency department users with psychological problems	San Francisco United States	Total sample n=252 Case management n=167 Usual care n=85	Patients randomized to case management received long-term clinical case management that included assessment, crisis intervention, individual and group supportive therapy, assistance in obtaining stable housing and income entitlements, linkage to medical care providers, referral to substance abuse services when needed, and ongoing assertive community outreach to maintain continuity of care with a maximum caseload of 15 patients.	Patients randomized to usual care were eligible to receive case management services at the conclusion of the 24-month study period.	Housing stability, Mental health, Substance use, Hospitalization, Income	Patients randomized to the case management condition showed significantly lower levels of homelessness, problematic substance use, hospitalization, unmet financial needs than those in usual care. No statistically significant differences were found for psychiatric symptoms
Toro et al. 1997[80]	N/A	Randomized control trial	18 months	Mentally ill homeless adults with children	Buffalo United States	Total sample size n=202 cases Intervention n=101 control n=101	**Demonstration Employment Project - Training and Housing (DEPTH) -** holistic approach that combines services concerned with job training - placement and locating permanent housing and support services ll targeted to the individual's specific needs and oriented toward the long term goal of helping the person escape homelessness. Central to DEPTH's services was intensive case management, offering access and linkage to services.	Control: Those in the no-treatment control group received none of the DEPTH's services but were free to seek whatever other services were available to them in the community during the follow-up period	Housing stability, mental health, substance use, income	Even though there was a dramatic improvement in terms of number of days homeless, no statistically significant between groups difference was found on this outcome or substance use, and income outcomes. There was a significant improvement favouring the intervention group in psychological symptoms

										measured by the Brief Psychiatric Rating Scale (BPRS)

Appendix 5.3: Characteristics of Included Studies (ACT):

Study ID	Publication related to this study	Study design	Study follow-up	Study population	Study setting	Sample size	Intervention	Comparator	Outcomes of interest	Findings
Clark et al. 2000 [55]	N/A	3-arm randomized control trial	24 months	Clients with serious and persistent mental illness who were at risk of being homeless	Portland, Oregon<br><br>United States	Total sample n=163<br>Consumer ACT n=57<br>Non-consumer ACT n=57<br>Usual care n=49	The ACT programs: Each team consisted of four full-time and one part-time case managers, one of whom was the team leader. Both teams shared a psychiatrist, a nurse practitioner, and a clinical director. The psychiatrist conducted initial psychiatric assessments, prescribed and monitored medication, and participated in treatment planning. The clinical director provided consultation to the two teams and handled administrative tasks. The **Consumer ACT** staff held a bachelor's degree, had more average experience in the mental health field, had more mental disorders themselves than the non-consumer team. The average caseload for the ACT team during the study period was 4.6 clients per case manager. **Non-consumer ACT**: Staff reported no diagnosable mental illness. The majority of the non-consumer-ACT staff held a master's degree. The average caseload for the ACT team during the study period was 5.4 clients per case manager.	The "Usual Care" control condition:<br>Most subjects assigned to usual care were served by one of four community mental health centers (CMHCs) and a number of smaller, more specialized agencies in the Portland metro area usual care agencies set up special, intensive, community-based programs to better serve these high-need clients in the community. The intensity of services in these programs rivaled the intensity of the ACT teams. Average caseload size for usual care overall was 26.9.	Hospitalization	No statistically significant between-group differences were found on homelessness status, number of participants hospitalized and number of participants visiting the emergency department over 24 months
Essock et al. 1998 [56]	N/A	Randomized control trial	18 months	Homeless clients who were high service users with serious mental disorders	Connecticut, Mas<br><br>United States	Total sample n=262<br>ACT n=131<br>SCM n=131	ACT: Mobile and multidisciplinary, included nurses and at least a half-time psychiatrist, and had relatively rich staffing ratios (one staff member for every five to seven clients). This staff provided coverage for two shifts; coverage for a third shift was provided either by ACT staff on beeper or a mobile crisis team with whom the ACT teams worked closely. The ACT teams served 50-71 clients who received case management, outpatient clinical services (such as medication management and group therapy), mobile outreach and crisis intervention services	SCM interventions: SCM case managers had caseloads of 25-30 clients. Those receiving SCM averaged one to two hours per month with their case manager. SCM clients had access to these same services, either as provided by their individual case manager or via specialized service providers to which the case manager arranged linkage. Clients from each condition had access to the same array of residential services, psychosocial clubs, crisis/respite programs, and vocational service providers	Mental health, Quality of life, Hospitalization	Participants receiving ACT reported 50% less days in the hospital and 30% more days in stable housing than those receiving standard case management services. However, ACT participants were not discharged more rapidly from hospital than those in the SCM group. Participants in the ACT group reported better improvements in general life satisfaction as well as personal safety, leisure activities, living situations, and frequency of contacts with friends subscales of Lehman's QOLI than those in the SCM group. No statistically significant between-group difference favouring the ACT group was found on mental health

										outcomes
Essock et al. 2006 [25]	N/A	Randomized control trial	3 years	Homeless adults with mental illness and an active substance use disorder	Connecticut, Mas<br><br>United States	Total sample n=198 ACT n=99 SCM n=99	Assertive Community Treatment (ACT): Mobile and multidisciplinary, included nurses and at least a half-time psychiatrist, and had relatively rich staffing ratios (one staff member for every five to seven clients). This staff provided coverage for two shifts; coverage for a third shift was provided either by ACT staff on beeper or a mobile crisis team with whom the ACT teams worked closely. The ACT teams served 50-71 clients who received case management, outpatient clinical services (such as medication management and group therapy), mobile outreach and crisis intervention services	Standard case management: Delivered at least some services in the community, had clinicians work with the clients' support systems, and vigorously addressed substance use disorders. Because clinicians in the standard clinical case management group had caseloads of approximately twice as many clients as clinicians in the assertive community treatment group, they provided fewer services directly.	Mental health, Quality of life, Hospitalization	No statistically significant between-group differences were found on quality of life or mental health outcomes. Participants receiving ACT services reported significantly less days in hospital than those receiving standard case management. Improvements in substance use and housing stability outcomes were inconsistent and depended on site
Fletcher et al. 2008 [57]	N/A	3-arm randomized control trial	30 months	Homeless adults with severe mental illness and a substance use disorder	St. Louis, MO<br><br>United states	Total sample n=191 [No description of allocation]	The IACT team had a substance abuse specialist on staff and provided outpatient substance abuse counseling and bi-weekly treatment groups.<br>The ACTO team referred clients to other community providers for outpatient or individual substance abuse services and to 12-step groups.	Participants assigned to SC were shown a list of community agencies that provided mental health and substance abuse treatment. Research staff provided these participants with information about treatment openings and assisted individuals in making their initial contact with an agency.	Housing stability, Mental health, Substance use	Participants in both IACT and ACTO groups reported more days in stable housing than those in the SC group. However, no significant differences between ACTO and IACT modalities were found. While both groups reported improvements in mental health, and substance use outcomes, no statistically significant between-group differences were found on any of the outcomes measured
Lehman et al. 1997 [58]	N/A	Randomized control trial	12 months	Homeless persons with mental illness	Baltimore, MD<br><br>United States	Total sample n=152 Intervention n=77 control n=75	The experimental condition was the ACT program, modeled after the ACT program first developed by Stein and Test. Each patient was assigned to a "mini-team" consisting of a clinical case manager (caseload, 10-12 patients), an attending psychiatrist, and a consumer advocate. The entire ACT team, including the consumer advocates, worked together in decision making and each staff member was knowledgeable about most of the patients. Team-work was fostered through daily sign-out rounds and twice-weekly treatment planning meetings.	The comparison condition consisted of services as usual in Baltimore. The public mental health system in Baltimore encompasses 7 community mental health centers operating under a nonprofit, private, local mental health authority, which was developed as part of the Robert Wood Johnson Foundation Program on Chronic Mental illness. Several community-based psychiatric inpatient and emergency facilities, including those affiliated with 2 major teaching institutions; provide acute inpatient and crisis-oriented care.	Housing stability, Mental health, Quality of life, Hospitalization	Participants receiving ACT reported significantly better clinical psychiatric symptoms, fewer emergency department visits and more number of days in community housing than those in the control group. No between-group differences were found on number of days homeless in shelters, objective quality of life or most of life satisfaction subscales (except general wellbeing and neighbourhood). The difference between groups on number of days homeless on the street favoured the ACT group and approached statistical significance.
Morse et al. 1992 [59]	N/A	3-arm Randomized control trial	12 months	Homeless people with serious psychiatric disorders	St. Louis, MO<br><br>United States	Total sample n=178 Treatment team n=52 Drop-in n=62 Outpatient	**Continuous treatment team:** included a "no-reject" policy, provision of community-based services for an unlimited time, and a flexible, individualized approach to address clients' multiple needs. Clinical case managers to work	**Outpatient treatment:** Traditional outpatient treatment was provided at a mental health clinic operated by the Missouri Department of Mental Health. The program offered psychotherapy, psychiatric medication, and	Housing stability, mental health, substance use, income	Participants in the Continuous treatment team reported better improvement on housing stability outcomes than those at the outpatient clinic who in turn reported better improvement than those at the drop-in centre. No

						n=64	intensively with clients, in a ratio of one staff member for every ten clients. In addition to outreach, service activities were targeted to three areas-individual change, environmental change, and support for bridging the gap between clients' needs and environmental resources and demands.<br><br>**Drop-in center**: Centers provided homeless people with respite from life on the street during the daytime, when the emergency shelters were closed, and offered food, clothing, showers, and some recreational opportunities such as card playing. Social workers were available to refer clients to social services; client-to-staff ratio was about 40 to 1.	assistance in obtaining social services.		statistically significant between group differences were found on mental health, substance use, or income outcomes.
Morse et al. 1997 [62]	Kenny et al. 2004 [63]	3-arm randomized control trial	18 months	Homeless mentally ill individuals	St. Louis, MO<br><br>United States	Total sample n=165 ACT and ACT with community workers n=105 BCM n=60	Assertive community treatment: Principles included intensive individualized treatment, responsibility for providing or coordinating all services needed by the client, persistent follow-up, and in vivo service delivery. No time limit was placed on treatment. The team conducted individual treatment activities, such as building a therapeutic alliance, linking clients with medication services, helping clients cope with symptoms and solve practical problems in daily living. The team also made interventions to improve clients social environment and resources and provided supportive services, such as monitoring medications, providing payee and money management services, and assisting with transportation.<br><br>Assertive Community Treatment with community workers: The approach operated similarly to the ACT only condition with one exception; clients were also assigned a paraprofessional community worker whose role was to assist with activities of daily living and to be available for leisure activities.	In the **broker case management** condition, the case manager's role was to develop an individualized service plan for the client, arrange for and purchase mental health and psychosocial services from various service providers, monitor the quality of purchased services and adjust the mix of services based on the client's changing needs.	Housing stability, Mental health, Substance use, Income	ACT participants reported better improvements in housing stability outcomes than those in the ACT with community workers groups, who in turn reported better improvements than those receiving broker case management services. When looking at the Brief Psychiatric Rating Scale (BPRS) and using Post hoc analysis, participants in both ACT conditions reported fewer symptoms in the areas of thought disorder and unusual activity than those receiving broker case management services. However, no significant between-group differences were found on anxiety-depression, hostility-suspicion, or self-esteem scales. Furthermore, no significant between-group differences were found on substance use or income outcomes.
Morse et al. 2006 [59]	Morse et al. 2008 [60] is a quasi-experimental	Randomized control trial	24 months	Homeless clients with severe mental illness and	St. Louis, MO	Total sample n=149 IACT n=46 ACTO n=54	**Integrated ACT** (and **New Integrated ACT** in Morse 2008) had a substance abuse specialist on staff and provided substance abuse	Participants assigned to the **standard care control** condition were shown a list of community agencies that provided mental	Housing stability, Mental health, Substance use	Participants receiving any of the ACT modalities (ACTO, IACT, NIACT) reported more days in stable housing than those in the

	follow-up for 18 months			substance use disorder	United States	SC n=49 + NIACT n=79 (Morse 2008)	services directly as part of the ACT team. These services included individual substance abuse counseling and bi-weekly treatment groups<br><br>**ACT only** team was instructed to refer clients to other community providers for outpatient or individual substance abuse services and to 12-step groups.	health and substance abuse treatment. Research staff also provided these participants with current information about openings at the various agencies and provided linkage assistance to help participants access services at these agencies.		standard control group. No differences were found between ACT modalities on this outcome. Even though participants receiving any of the interventions reported improvements over time in mental health and substance use outcomes, no statistically significant differences favouring ACT modalities over standard care were found for any of the the outcomes measured

Appendix 4.4: Characteristics of Included Studies (CTI):

Study ID	Publication related to this study	Study design	Study follow-up	Study population	Study setting	Sample size	Intervention	Comparator	Outcomes of interest	Findings
De Vet et al. 2017 [81]	N/A	Randomized control trial	up to 9 months	Homeless adults who had resided in a shelter for less than 14 months and were moving to housing	Netherlands	Total sample n=183 Intervention n=94 control n=89	Critical Time Intervention (CTI). Time-limited, strength-based intervention for vulnerable people, which bridges the gap between services during times of transition, generally consisted of discharge planning and referral services and access to a range of community-based services. The CTI worker provides practical and emotional support and helps to develop and strengthen links with community resources, creating a network that will continue to provide support long after CTI has ended. Delivered in three phases of 3 months: transition to the community (phase 1), try-out (phase 2), and transfer of care (phase 3	Care-as-usual	Housing stability, Mental health, Quality of life, Substance use	No statistically significant difference was found on number of days rehoused. Even though participants randomized to the CTI group reported better mental health, quality of life, and substance use improvements, these differences were small and not statistically significant.
Herman et al. 2011 [82]	Tomita et al. 2012 [83]	Randomized control trial	18 months	Homeless mentally ill participants after hospital discharge	New York<br><br>United States	Total sample n=150 CTI n=77 Control n=73	CTI: Nine-month case management program delivered in three phases of 3 months each; Phase 1: "Transition to the community": providing intensive support and assessing the resources that exist for the transition of care to community providers. The CTI worker generally makes detailed arrangements in only the handful of areas seen as most critical for community survival of that individual. Phase 2: "Try out": CTI worker can focus on assessing the degree to which this support system is functioning as planned. In this phase, the worker will intervene only when modification in the system is needed or when a crisis occurs. Phase 3: "Transfer of care": focuses	"Usual" community-based services depending on the individual's needs, preferences and living situation. These services usually included various types of case management and clinical treatment.	Housing stability, Hospitalization	When looking at the final three follow-up intervals, using an intent-to-treat analysis, and adjusting for baseline homelessness, those assigned to CTI reported a significant five-fold reduction in the odds of homelessness as well as a statistically significant reduction in odds of rehospitalization and number of nights homeless compared to the usual care group. Furthermore, CTI clients had significantly lower frequency and proportion of rehospitalization nights compared to control group clients. When examining these outcomes over the entire 18 months study period, assignment to the intervention was associated with a

							on completing the transfer of responsibility to community resources that will provide long-term support			substantial, but marginally significant, decrease in homelessness odds, and a statistically significant reduction in number of nights homeless favouring the intervention group.
Lako et al., 2018[84]	N/A	Randomized control trial	9 months	Women over the age of 18 staying at a shelter due to intimate partner violence	Multi-city<br><br>The Netherlands	Total sample n=136 Intervention n=70 Usual care n=66	CTI, consisting of three phases: (1) transition to the community; (2) try-out; (3) transfer of care.	Care-as-usual: Most organizations provided support during regular meetings (1-3 h per week) for 13-52 weeks. All organizations employed a strengths based approach	Quality of life, mental health	There was no significant between-group difference in QoL during follow-up. Women in the experimental group experienced significantly less symptoms of PTSD during follow-up. No between-group differences were found for symptoms of depression or psychological distress.
Shinn et al. 2015 [85]	Samuels et al. 2015[86]	Randomized control trial	24 months	Shelter-dwelling families with child. A mother diagnosed with mental illness or substance use	Westchester County, New York<br><br>United States	Total sample n=200 Intervention n=97 Usual care n=103	Family Critical Time Intervention (FCTI): A community-based service model for families using homeless shelters. Multidisciplinary teams help connect the family with social services and form supportive relationships with families and friends. This FCTI targets the critical time of transition from the shelter to housing in the community through three phases:<br>Transition to Community Phase: A family arrives in the shelter and is assessed thoroughly by a case manager, who then works intensely with the mother, up to three times per week. Try-Out Phase: The case manager reduces contact during this phase, but still supports the family through adjusting the support systems as they move into the community. Transfer to Care: This phase involves long-term linkage with community-based services to allow the family to take full responsibility for accessing services.	Case management with a caseload of 24 families or more. The families, after meeting caseworker standards for housing readiness, had access to scattered site subsidized housing.	Housing stability, Mental health	Families receiving FCTI reported higher rates of time spent in conventional housing in the community than the control group. However, when the FCTI services ended at 9 months, housing findings converged. FCTI showed a reduction in both internalizing and externalizing behaviours of children aged 1.5-5 years. However, no benefits were found for children aged 6-10 years, and only a significant reduction in externalizing behaviour was reported by mothers of children aged 11-16 years. No benefits on self-reported externalizing behaviours were found for this age group.
Susser et al. 1997 [87]	Jones et al. 1994, Jones et al. 2003, Lennon et al. 2005, Herman et al. 2000[88-91]	Randomized control trial	18 months	Homeless mentally ill men	New York<br><br>United States	Total sample n=96 CTI n=48 TAU n=48	CTI: To implement the first component of CTI, the clinical team devised a plan for the transfer of care from the shelter to other formal and informal supports. The plan focused on specific areas of potential discontinuity that were related to the risk of homelessness for that individual-for instance, medication adherence and/or money management. Each man was then assigned to a CTI worker to implement the plan. CTI work entailed visiting the family home or community residence, being present	TAU: For usual services, the men were referred to mental health and rehabilitation programs that were generally of high quality. Following the usual model of discharge from an institution, the staff of the on-site shelter psychiatry program were available to these agencies for consultation on request, but they did not actively seek a role in the client's care after discharge. The men were also referred as needed to community agencies for substance abuse, general health,	Housing stability, Mental health	Over 18 months, compared to usual care, participants receiving CTI reported better housing outcomes including fewer number of days homeless, lower risk of a major homeless episode, higher number of nonhomeless nights and lower odds of extended homelessness. A follow-up reported that assignment to CTI was associated with significant decreases in negative psychiatric symptoms at 6 months. In another follow-up, participants receiving CTI were slightly more likely to have hospital stays and

							at appointments, and locating patients and giving advice in times of crisis. To implement the second component of CTI, during the first 2 weeks after discharge the CTI worker spent time with the client in the community and observed his physical and social surroundings and daily habits. Subsequent support was individually tailored	income support, education, legal advocacy, and other services.		hospitalization nights than the control group. However, both reported similar average length of hospital stays. Income earning were low and the difference between groups was not statistically significant

Appendix 4.5: Summary of characteristics of included cost or cost-effectiveness studies

Study	Study Design	Population	Intervention/Comparator	Resource Required	Cost/Cost-effectiveness
Aubry et al. 2016[93]	Cost analysis conducted alongside an RCT. No perspective of analysis specified. Cost measured in C$	Adult participants (18+) who had a high need for treatment: absolutely homeless/ precariously housed, had a current mental disorder, not receiving ACT or ICM, and legal status as a Canadian citizen, landed immigrant, or refugee claimant.	Housing First programs with ACT<br>Treatment as usual: any housing and community support services other than for the Housing First program	Annual cost of housing First with ACT services was C$22,257 per participant	Housing First Program led to a reduction in the average cost of C$21,367 per person per year, this cost offset was associated with office visits, hospitalizations, emergency shelter visits, home visits and incarceration.<br>The savings gained by Housing First did not fully offset its cost.
Clark et al. 1998[94]	Cost-effectiveness analysis conducted alongside an RCT, societal perspective. Costs were reported in 1995 US$	Persons with schizophrenia, schizoaffective disorder, or bipolar disorder and a concurrent substance use disorder	Specialized treatment for dual disorders in an ACT team<br>SCM program with targeted substance	Mental health (in and outpatient), general health (in and outpatient), legal, and community services (shelter, public guardian, fire and ambulance, family)	ACT more effective and less costly than SCM in both substance abuse and quality of life comparisons.<br>The relationship between the treatment received and benefit to cost ratio was curvilinear. During earlier periods SCM produced better outcomes per $10,000 invested than did ACT. During the final year of the study, ACT produced substantially better outcomes per $10,000 than SCM.
Essock et al. 1998[25]	Cost-effectiveness analysis based on an RCT, societal perspective. Costs were reported in 1992 US$.	Clients of the Connecticut Department of Mental Health with serious mental disorders who were heavy users of acute services at the time of recruitment and had difficulty meeting the demands of everyday life in the community.	ACT<br>SCM	Clients on ACT teams had a mean of nine hours of face-to-face contact with staff per month, whereas those receiving SCM had a mean of one to two hours per month with their case manager.<br><br>ACT clients were served by a team and saw an average of five to eight staff per month compared to the single case manager seen by SCM clients.<br><br>Clients assigned to ACT teams received case management, outpatient clinical services, mobile outreach and crisis intervention services from ACT team members.	The mean annual cost to society for a client in the ACT condition was $33,473 (SD=32,838), compared to $35,656 (SD= 39,446) for those in the SCM condition.<br>ACT clients spent significantly more time in the community than did SCM clients. The mean number of community days during the target year was 301 (SD=115.5) for ACT clients and 261 (SD=144.8) for SCM clients (p<.05).<br>ACT led to fewer homeless days and lower societal costs than SCM. For the ACT group, an effectiveness of 301 community days was divided by $33.5 thousand in societal costs to equal nine community days expenditure; for the SCM group, 261 community days/$35.7 thousand in societal costs=7.3 community days per $1,000 expenditure.

				SCM clients had access to these same services,<br>either as provided by their individual case manager or via specialized service providers to which the case manager arranged	ACT was more cost-effective for those clients who were hospitalized at study entry
Lehman et al. 1999[95]	A cost-effectiveness based on a published RCT, a health care payer's perspective. Inclusive of direct treatment costs. Currency year was not reported.	Homeless persons with severe and persistent mental illness in Baltimore, Maryland	ACT<br>Usual community services available in Baltimore.	Intervention cost<br><br>Health service use: outpatient, inpatient, emergency room and rehabilitation	Mean ACT cost per case was $8,244.<br>The overall average cost per ACT patient was $15,732 less than the cost per usual-care patient.<br>The median total cost per case for the ACT patients was $26,193 compared with $33, 827 for the usual care patients. However, the total per case cost did not reach statistical significance.<br><br>ACT led to lower costs and more day housed than usual care. The cost-effectiveness ratios were $241 per day housed for the ACT patients compared with $415 per day housed for the usual care patients. In other words, each day of stable housing was achieved for $174 less in direct treatment costs by the ACT program than by usual care, a relative efficiency
Morse et al. 2006[59]	A cost- consequence analysis from a societal perspective. The study was based on a randomized study.	Individuals with dual disorders (i.e., individuals<br>with co-occurring severe mental illness and substance use disorders) who were<br>homeless	Integrated ACT (IACT)<br>ACT only (ACTO)<br>Standard care (control group)	Outpatient costs (direct treatment, mental health, substance abuse physical health, psychological rehabilitation center)<br>Inpatient costs (mental health, substance abuse, physical health, emergency shelter)	IACT and control groups had significantly lower total costs than the ACTO condition, but there was no significant difference in total costs between IACT and control groups.<br><br>Clients in the ACTO and IACT were significantly more satisfied with their treatment and had significant more days in stable housing than those in the control group.
Wolff et al. 1997[96]	A cost-consequence analysis based on an RCT. Cost data were presented in 1992 US$.	Individuals with severe mental illness who were at risk for homelessness	ACT alone<br>ACT with community workers<br>Brokered case management (purchase of services)	Mental health inpatient<br>Mental health outpatient<br>Physical health inpatient<br>Physical health outpatient<br>Vocational general equivalency<br>Residential: emergency shelter, housing subsidy<br>Maintenance: supplemental security income, food stamps	The total costs for ACT only, ACT with community workers, and brokered case management were $49,510, $39,913, and $45,076, respectively. There was no statistically different in total costs across 3 groups.<br><br>The ACT with community workers (and the effect of ACT only) were statistically associated with better more contacts with

					case managers, improved client satisfaction, and fewer mental health symptoms.
Nyamathi et al. 2016[50]	A cost analysis based on an RCT. Currency year was not reported.	Men recently released from county jails and state prisons	Intensive peer coach and nurse case-managed (PC-NCM) program<br>Intermediate peer-coaching (PC) program with brief nurse counseling<br>Usual care (UC) program	Actual cash expenditures used to procure vaccines or paid directly to participants as incentives: the cost of acquiring vaccines, cash incentives for urine analysis, and cash payment for baseline, and two follow-up assessments.<br><br>Salaries and benefits of the staff who were directly involved in the delivery of the services	The amount of cash spent on program activities was about the same for all three groups of participants: 32,583 for the PC-NCM participants (M=$167.09; SD=$79.51), $33,375 for PC (M=$170.28; SD=$76.20), and $33,293 for UC (M=159.30; SD=$76.61).<br>The PC-NCM group consumed the most staff time (more than half or 54 % of the total recorded staff time), followed by PC group with about 44 % of the staff time, while the UC group used the least staff time, with only 2.11 % of the staff time.<br>On an annualized basis, participants of the PC-NCM group on average consumed $593.26; participants in the PC group on average consumed $488.92; and participants of the UC group consumed $59.92.
Okin et al. 2000[92]	A cost-benefit analysis based on a prospective, pre-post study, a hospital's perspective. No statistical approach was used to adjust for confounding factors. Cost data were reported in 1997 US$.	Adult who used the ED five times or more in 12 months	Before and after CM intervention	Costs of medical and psychiatric inpatient, outpatient and emergency services<br><br>Costs of physicians' professional services<br><br>Ambulance costs<br><br>Case management	The median total hospital service cost decreased from $21,022 in the year before case management enrollment to $14,910 in the year after enrollment (median change= $22,406, P=.06, 95% CI: -$6,361 to -$430).<br><br>When the total cost of case management services to the 53 patients, calculated at $296,738, was subtracted from the $429,464 savings realized in other hospital services, there was a net cost saving of 132,726, indicating that for each dollar invested in the case management program, there was a $1.44 reduction in other hospital
Rosenheck et al. 2003[98]	Cost-effective analyses based on an RCT. Costs were evaluated from the perspectives of the VA, the total health care system (VA and non-VA), the government (for taxpayers) and society.	Homeless veterans with psychiatric and/or substance disorders or both	HUD-VASH program with housing vouchers<br>CM only without housing vouchers<br>Standard VA care	Outpatient care costs: mental health care, medical-surgical care, homeless case management<br>Inpatient and residential care costs: mental health care, medical-surgical care, residential care<br>Non-health costs: shelter, incarceration, administrative cost of section 8 vouchers and earned income (productivity)	Over a 3-year period, total societal cost for CM only group was $3,500 higher than standard VA care.<br>CM only group had slightly more days housed than standard VA care (50.8 vs 47.6 days).<br>From a societal perspective, each additional day housed among CM only clients costed $1,094.
Shumway et al. 2008[79]	A cost-effectiveness analysis based on an RCT, hospital's perspective. Cost data were presented in 2001 US$. No	Frequent users of the emergency department (made 5 or more visits to the ED in the prior 12 months) and had psychosocial problem	Comprehensive clinical CM<br>Usual care	ED visits<br>Medical inpatient admissions<br>Medical inpatient days<br>Psychiatric emergency visits	Emergency department costs were significantly lower among CM patients than among usual care patients. The costs of medical inpatient services, psychiatric

	cost-effective ratio was reported. This study should be categorized as a cost analysis.	including homelessness, alcohol use, lack of health insurance and social security income, and financial need.		Psychiatric inpatient visits<br>Psychiatric inpatient days<br>Medical outpatient visits<br>Physician visits<br>ED case management cost	emergency services,<br><br>Psychiatric inpatient services, medical outpatient services, and physicians' professional fees did not differ between CM and usual care patients.<br><br>When the costs of the ED Case Management Program were considered, total hospital costs were similar for CM and usual care patients.
Stergiopoulos et al. 2015[97]	A cost analysis based on an RCT, society perspective. Cost data were presented in 2010/11 C$.	Participants of community agencies and institutions serving homeless individuals who met the following criterial: 1) legal age of majority; 2) absolutely homeless or precariously housed; and 3) presence of a mental illness, with or without a concurrent<br>substance use disorder, as evaluated using the Mini International<br>Neuropsychiatric Interview.	Scattered-site supportive housing with mobile, off-site ICM services, offering rapid, low-barrier permanent housing in independent units with supports fostering participant empowerment, choice, personalized goals, hope, and resilience<br>Usual care: access to existing housing and support services in their communities	Program cost: contributions by private donors and government sources, welfare and disability payments, capital cost<br>Residential health, social and justice services: hospitalization, nursing and long-term care facilities, psychiatric rehabilitation residential program, addictions treatment or residential recovery, detox facilities, crisis housing, SROs with supports, emergency shelters, jails or prisons, corrections half-way house, all other housing<br>Non-residential services: outpatient consultations, ED visits, ambulance transports, crisis lines, mobile crisis teams, day (drop-in) centers, community meal centers or meal programs, food banks, community-based provider visits, police contacts, arrests, detentions, court appearances<br>Housing first intervention: rent supplements and housing teams, ICM teams<br>Government assistance less earnings: social assistance, disability and other benefits	The average annual cost of supportive housing with ICM services was C$14,177 per participant, resulting in an average net cost offset of C$4,849 per participant per year, or 34% of the cost of the intervention.
Jones et al. 2003 [89]	Cost-effectiveness analysis alongside an RCT, societal perspective. Cost data were presented in 1992 US$.	Enrollees of a psychiatric program in a men's shelter in New York City	CTI<br>Usual care	Earned income<br>Cost of the intervention<br>Outpatient mental health care/medical care<br>Acute services<br>Substance abuse services<br>Supported/other housing<br>Shelter<br>Criminal justice<br>Family donations<br>Public transfers	Over 18 months, the total cost incurred by CTI clients was numerically higher than those receiving usual care ($52,374 vs $51,649).<br>CTI significantly reduced nonhomeless nights compared to usual care (508 vs 450, p=.01).<br>Over 18 months, the CTI was considered cost-effective if the society were willing to pay $152 per nonhomeless night.
