# Supplementary material for: The effectiveness of case management interventions for the homeless, vulnerably housed and persons with lived experience: A systematic review
Source: PLoS One. 2020 Apr 9;15(4):e0230896. doi: 10.1371/journal.pone.0230896 (PMC7313544; doi:10.1371/journal.pone.0230896)
Supplement: S5 File — (PDF) [file pone.0230896.s005.pdf]

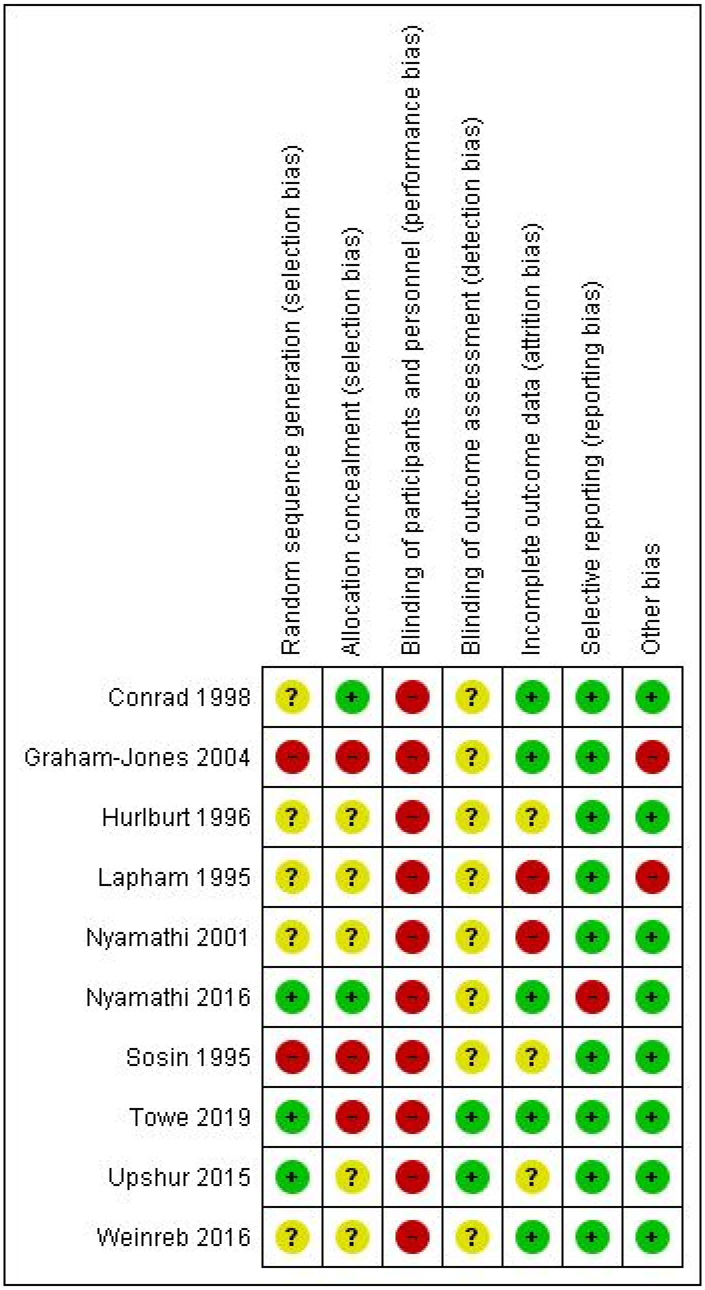

Risk-of-bias summary: review authors' judgments about each risk-of-bias item for each included SCM study

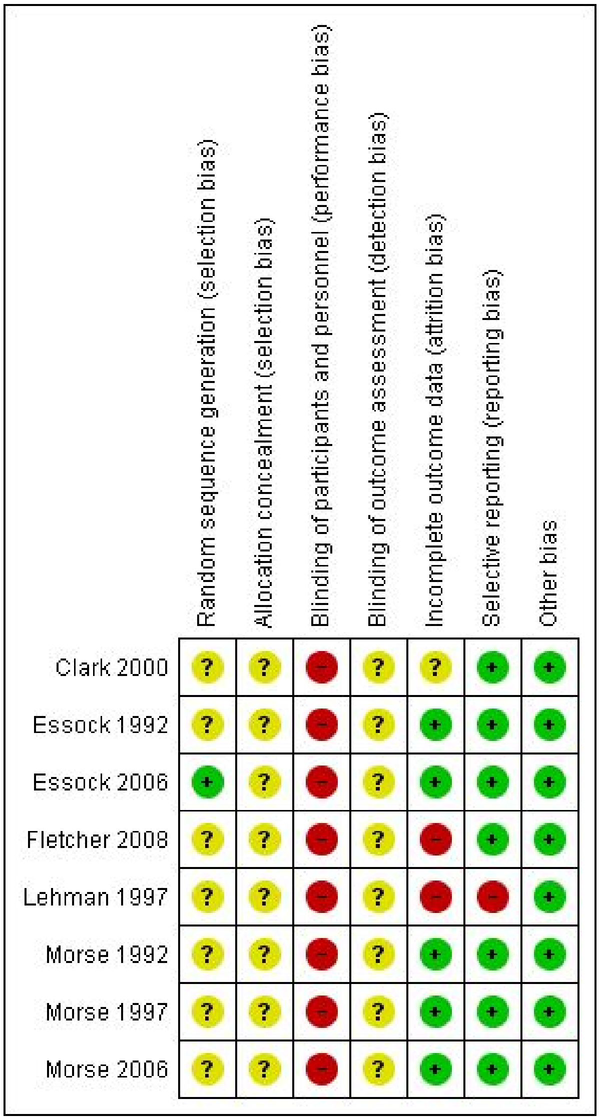

Risk-of-bias summary: review authors' judgments about each risk-of-bias item for each included ACT study

	Random sequence generation (selection bias)	Allocation concealment (selection bias)	Blinding of participants and personnel (performance bias)	Blinding of outcome assessment (detection bias)	Incomplete outcome data (attrition bias)	Selective reporting (reporting bias)	Other bias
Braucht 1995	+	?	−	?	+	+	+
Burnam 1995	?	?	−	?	−	+	+
Cauce 1994	+	?	−	?	?	+	+
Clark 2003	−	−	−	?	−	+	+
Cox 1998	?	?	−	?	−	+	+
Felton 1995	−	−	−	+	+	+	+
Grace 2014	−	−	−	?	−	−	−
Korr 1995	?	?	−	?	−	+	+
Malte 2017	+	+	−	−	+	+	−
Marshall 1995	+	?	−	?	?	+	+
Orwin 1994	?	?	−	−	−	+	−
Rosenblum 2002	−	−	−	?	−	+	+
Schutt 2009	?	?	−	?	?	?	?
Shern 2000	?	?	−	?	+	+	+
Shumway 2008	+	?	−	?	+	+	+
Stahler 1995	?	?	−	?	+	+	+
Toro 1997	?	+	−	−	+	+	+

Risk-of-bias summary: review authors' judgments about each risk-of-bias item for each included ICM study

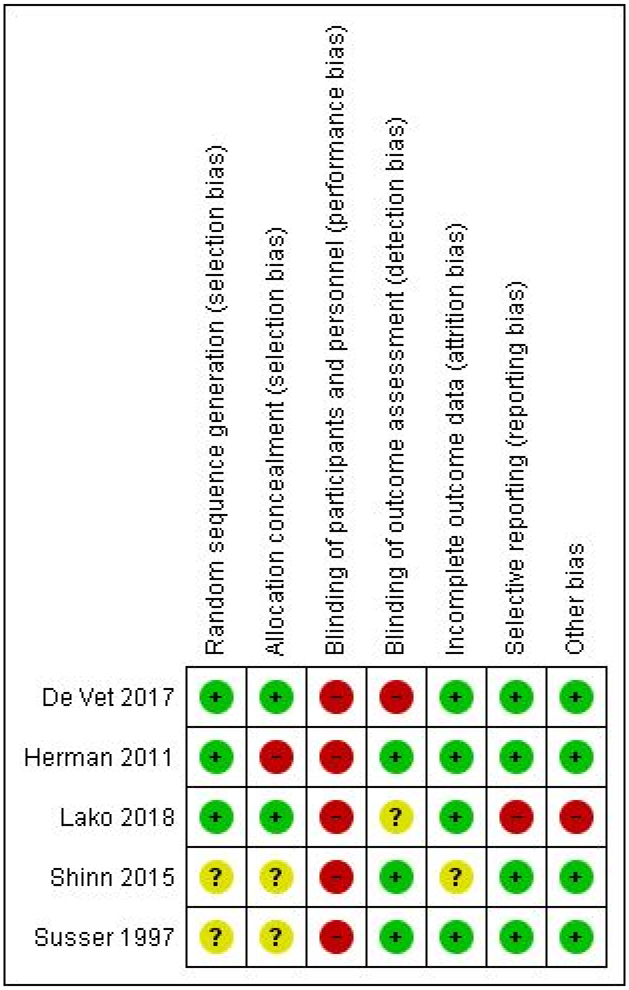

Risk-of-bias summary: review authors' judgments about each risk-of-bias item for each included CTI study
