# Supplementary material for: The effectiveness of case management interventions for the homeless, vulnerably housed and persons with lived experience: A systematic review
Source: PLoS One. 2020 Apr 9;15(4):e0230896. doi: 10.1371/journal.pone.0230896 (PMC7313544; doi:10.1371/journal.pone.0230896)
Supplement: S6 File — (PDF) [file pone.0230896.s006.pdf]

The Effectiveness of Case-Management Interventions for the Homeless, Vulnerably Housed and Persons with Lived Experience: A Systematic Review and Meta-Analysis.

Appendix VI: GRADE Tables

Appendix 5.1: SCM GRADE Evidence Profiles

Certainty assessment							№ of patients		Effect		Certainty	Importance
№ of studies	Study design	Risk of bias	Inconsistency	Indirectness	Imprecision	Other considerations	Case Management	Comparison	Relative (95% CI)	Absolute (95% CI)		
**Outcome: Housing Stability**												
Percentage of participants homeless (Shumway 2008) (follow up: 24 months)												
1	randomised trials	serious [a]	not serious	not serious	Serious [b]	none	38/141 (27.0%)	21/85 (24.7%)	**OR 1.12** (0.61 to 2.08)	**26 more per 1,000** (from 101 fewer to 177 more)	⊕⊕◯◯ LOW	CRITICAL
Percentage of participants who lived in one residence in the last 3 months (Upshur 2015) (follow up: 6 months)												
1	randomised trials	serious [g]	not serious	not serious	Serious [b]	none	9/40 (22.5%)	16/36 (44.4%)	**OR 0.36 (0.13 to 0.97)**	**221 fewer per 1,000 (from 350 fewer to 8 fewer)**	⊕⊕◯◯ LOW	CRITICAL
Percentage of participants who lived in three residences or more in the last 3 months (Upshur 2015) (follow up: 6 months)												
1	randomised trials	serious [g]	not serious	not serious	Serious [b]	none	12/40 (30.0%)	3/36 (8.3%)	**OR 4.71 (1.20 to 18.39)**	**216 more per 1,000 (from 15 more to 542 more)**	⊕⊕◯◯ LOW	CRITICAL
Mean Number of days in stable residence (Sosin 1995) (follow up: 6 months)												
1	randomised trials	serious [a]	not serious	not serious	Serious [c]	none	-	-	**The case management intervention increases residential stability by a statistically significant 9 days (8.650; t=2.35; p<0.05)**		⊕⊕◯◯ LOW	CRITICAL
**Outcome: Mental Health**												
Percentage of participants with positive depression symptoms (Upshur 2015) (follow up: 6 months)												
1	randomised trials	serious [g]	not serious	not serious	Serious [b]	none	12/37 (32.4%	20/36 (55.6%)	**OR 0.38 (0.14 to 0.99)**	**234 fewer per 1,000 (from 407 fewer to 2 fewer)**	⊕⊕◯◯ LOW	CRITICAL
**Outcome: Quality of Life**												
Change in overall life fulfillment using the Life Fulfilment Scale (LFS) (Graham-Jones 2004) (follow-up: 3 months)												
1	Observational studies	serious [a]	not serious	not serious	Serious [c]	none	75 (Health centre: 22; Outreach: 53)	42	Control: 0.3 +/- 23.6<br>Health centre: 13.3 +/- 18.8<br>Outreach: 16.9 +/- 20.5<br>Analysis of variance (P < 0.001): significantly more improvement (Bonferroni-corrected P < 0.05) in the outreach advocacy group compared with the control group		⊕◯◯◯ VERY LOW	CRITICAL
**Outcome: Substance Use**												
Problematic alcohol use for participants with 5-11 emergency department visits (Shumway 2008) (follow up:6 months)												

1	randomised trials	serious [a]	not serious	not serious	Serious [b]	none	18/78 (23.1%)	19/40 (47.5%)	OR 0.33<br>(0.14 to 0.74)	245 fewer per 1,000<br>(from 363 fewer to 74 fewer)	⊕⊕◯◯<br>LOW	CRITICAL
Problematic alcohol use for participants with 12 or more emergency department visits (Shumway 2008) (follow up:6 months)												
1	randomised trials	serious [a]	not serious	not serious	Serious [b]	none	28/88 (31.8%)	25/45 (55.6%)	OR 0.37<br>(0.17 to 0.78)	239 fewer per 1,000<br>(from 380 fewer to 62 fewer)	⊕⊕◯◯<br>LOW	CRITICAL
Number of days participants used other drugs in the last 3 months (Upshur 2015) (follow up: 6 months)												
1	randomised trials	serious [g]	not serious	not serious	Serious [c]	none	40	36	-	MD 12.9 more<br>(3.26 more to 22.53 more)	⊕⊕◯◯<br>LOW	CRITICAL
Average days of alcohol and drug consumption (Sosin 1995) (follow up: 12 months)												
1	randomised trials	serious [a]	not serious	not serious	Serious [c]	none	-	-	The case management only intervention decreased the reported average days of alcohol and drug consumption by a modest, but significant 2.5 days (t=-2.01; p<0.05) Sub-analysis: Substance consumption was lower for subjects who have higher levels of self-esteem (-0.305; t=-3.24; p<0.01) and lower levels of anxiety (0.221; t=2.25; p<0.05), as well as, higher levels of depression (-0.657; t=-2.03; p<0.05) at baseline. Consumption was higher for subjects with greater involvement with the legal system (8.028; t=2.96; p<0.01).		⊕⊕◯◯<br>LOW	CRITICAL
**Outcome: Employment**												
Employment problems (Conrad 1998) (follow-up: 24 months)												
1	Randomised trials	Serious [d]	Not serious	Not serious	Serious [e]	none	178	180	Both groups experienced fewer employment problems with time. The experimental group effects were significantly better during the entire 2-year period (p=0.04), [no further data]		⊕⊕◯◯<br>LOW	CRITICAL
Employment status "currently employed" (Weinreb 2016) (follow-up: 6 months)												
1	Randomised trials	Serious [d]	Not serious	Not serious	Serious [b]	none	7/21 (33.3%)	2/13 (15.4%)	**OR 2.75**<br>(0.47 to 15.96)	**179 more per 1,000**<br>(from 75 fewer to 590 more)	⊕⊕◯◯<br>LOW	CRITICAL
Employment status "part time or full time" (Nyamathi 2016) (follow-up: 12 months)												
1	Randomised trials	Serious [f]	Not serious	Not serious	Serious [b]	none	53/166 (31.9%)	63/186 (33.9%)	**OR 0.92**<br>(0.59 to 1.43)	**18 fewer per 1,000**<br>(from 107 fewer to 84 more)	⊕⊕◯◯<br>LOW	CRITICAL
**Outcome: Income**												
Percentage of participants with no social security income (SSI/SSA/SSD) (Prior ED visits: ≥12 group) (Shumway 2008) (follow up: 18 months)												
1	randomised trials	serious [a]	not serious	not serious	Serious [b]	none	23/58 (39.7%)	20/27 (74.1%)	**OR 0.23**<br>**(0.08 to 0.63)**	**344 fewer per 1,000**<br>**(from 555 fewer to 98 fewer)**	⊕⊕◯◯<br>LOW	CRITICAL

Explanations

a. High risk of performance bias, and unclear risk of detection bias
b. Too few events (<300)
c. Too few participants (<400)
d. Unclear risk of selection bias and detection bias, high risk for performance bias.
e. Point estimates and confidence intervals not provided
f. High risk of performance bias and reporting bias
g. Unclear risk of selection bias and attrition bias, high risk for performance bias.

Appendix 5.2: ICM GRADE Evidence Profile

Certainty assessment							№ of patients		Effect		Certainty	Importance
№ of studies	Study design	Risk of bias	Inconsistency	Indirectness	Imprecision	Other considerations	Case Management	Comparison	Relative (95% CI)	Absolute (95% CI)		
**Outcome: Housing Stability**												
Number of days homeless (Cox 1998, Grace 2014, Toro 1997) (Follow up: 13+ months)												
3	Randomised trials	Very serious [a]	Not serious	Not serious	Not serious	none	358	308	-	SMD **0.22 fewer** (0.4 fewer to 0.03 fewer)	⊕⊕◯◯ LOW	CRITICAL
Proportion of participants housed (Korr 1996) (Follow-up: 6 months)												
1	Randomised trials	Serious [b]	Not serious	Not serious	Serious [c]	none	36/48 (75%)	15/44 (34.1%)	**OR 5.80** (2.35 to 14.31)	**409 more per 1,000** (from 208 more to 540 more)	⊕⊕◯◯ LOW	CRITICAL
Time spent on the street (Shern 2000) (Follow-up: 24 months)												
1	Randomised trials	Serious [d]	Not serious	Not serious	Serious [e]	none	91	77	-	MD **26.71 fewer** (39.21 fewer to 14.2 fewer)	⊕⊕◯◯ LOW	CRITICAL
Time spent in shelters (Shern 2000) (Follow-up: 24 months)												
1	Randomised trials	Serious [d]	Not serious	Not serious	Serious [e]	none	91	77	-	MD **20.29 more** (13.38 more to 27.19 more)	⊕⊕◯◯ LOW	CRITICAL
Time spent in community housing (Shern 2000) (Follow-up:24 months)												
1	Randomised trials	Serious [d]	Not serious	Not serious	Serious [e]	none	91	77	-	MD **11.07 more** (1.52 more to 20.61 more)	⊕⊕◯◯ LOW	CRITICAL
**Outcome: Mental Health**												
Psychiatric symptoms using the Manchester Scale (Marshall 1995) (Follow-up: 14 months)												
1	randomised trials	Serious [f]	not serious	not serious	serious [e]	none	40	40	-	MD **0.75 higher** (1 lower to 2.5 higher)	⊕⊕◯◯ LOW	CRITICAL
Psychiatric symptoms using the SCL-90-R (Toro 1997) (Follow-up: 18 months)												
1	randomised trials	Serious [g]	not serious	not serious	Serious [e]	none	54	51	-	MD **0.19 fewer** (0.37 fewer to 0.006 fewer)	⊕⊕◯◯ LOW	CRITICAL
Psychiatric symptoms using the Colorado Symptom Index (Shern 2000) (Follow-up: 24 months)												
1	randomised trials	serious [d]	not serious	not serious	serious [e]	none	97	77	-	MD **0.32 fewer** (0.53 fewer to 0.1 fewer)	⊕⊕◯◯ LOW	CRITICAL
**Outcome: Substance Use**												
Number of days of alcohol use in the previous 30 days (Cox 1998) (follow up: 18 months)												
1	randomised trials	Serious [h]	not serious	not serious	not serious [e]	none	108	85	-	MD **4.0 fewer** (7.41 fewer to 0.58 fewer)	⊕⊕◯◯ LOW	IMPORTANT
Addiction Severity Index (ASI) alcohol composite score (Cox 1998) (follow-up: 6 months)												
1	randomised trials	Serious [h]	not serious	not serious	not serious [e]	none	108	85	-	MD **0.08 lower** (0.15 lower to 0.0009 lower)	⊕⊕◯◯ LOW	IMPORTANT
**Outcome: Quality of Life**												

Quality of life using Lehman's QOLI (Marshall 1995) (Follow-up: 14 months)												
1	randomised trials	serious [f]	not serious	not serious	serious [e]	none	40	40	-	MD **0.0** (0.42 lower to 0.42 higher)	⊕⊕◯◯ LOW	CRITICAL
Life satisfaction in 7 life areas using Lehman's QOLI (Shern 2000) (Follow-up: 24 months)												
1	randomised trials	serious [g]	not serious	not serious	serious [e]	none	91	77	Individuals in the experimental condition reported consistently greater improvement in life satisfaction than their peers in the control group in 6 of the 7 life areas (Overall, p=0.001; Leisure, p=0.027; Financial, p=0.001; Safety, p=0.005; Health, p=0.006; Family, p=0.005; Social, p=0.56).		⊕⊕◯◯ LOW	CRITICAL
**Outcome: Income**												
Public income (US$) in the last 30 days (Cox 1998) (Follow-up: 18 months)												
1	randomised trials	Serious[h]	not serious	not serious	not serious [e]	none	105	85	-	MD **89 higher** (7.59 higher to 170.4 higher)	⊕⊕◯◯ LOW	IMPORTANT
Income "from other sources" (Toro 1997) (Follow-up: 12 months)												
1	randomised trials	Serious[g]	not serious	not serious	Serious [e]	none	54	51	-	MD **901 higher** (110.99 higher to 1691 higher)	⊕⊕◯◯ LOW	IMPORTANT

CI: Confidence interval; **SMD:** Standardised mean difference; **OR:** Odds ratio; **MD:** Mean difference

Explanations

a. Two trials with unclear selection bias and one trial with high risk for selection bias (non randomised). Two trials with unclear detection bias, one trial with high risk of detection bias. Two studies with high risk of attrition bias.
b. Unclear risk of selection bias, high risk of performance bias, unclear risk of detection bias, and high risk of attrition bias
c. Small sample size, <100 events, wide confidence interval
d. Unclear selection bias, unclear detection bias, and high risk for performance bias
e.Small sample size, <300 participants
f.. Unclear risk of selection bias (allocation concealment), detection bias, and attrition bias. High risk of performance bias
g. Unclear risk of selection bias, high risk of performance bias and detection bias
h. Unclear risk of selection and detection bias, and a high risk of attrition bias

Appendix 5.3: ACT GRADE Evidence Profile

Certainty assessment							№ of patients		Effect		Certainty	Importance
№ of studies	Study design	Risk of bias	Inconsistency	Indirectness	Imprecision	Other considerations	Case Management	Comparison	Relative (95% CI)	Absolute (95% CI)		
**Outcome: Housing Stability**												
Number of days homeless on streets (Lehman 1997) (Follow up: 12 months)												
1	Randomised trials	Very serious [a]	Not serious	Not serious	Serious [b]	none	77	75	-	MD **14.2 fewer** (28.75 fewer to 0.35 more)	⊕◯◯◯ VERY LOW	CRITICAL
Number of days homeless in the previous month (Morse 1992) (Follow-up: 12 months)												
1	Randomised trials	Serious [c]	Not serious	Not serious	Serious [b]	none	52	62	-	MD **8.11 days fewer** (12.32 fewer to 3.89 fewer)	⊕⊕◯◯ LOW	CRITICAL
Number of days homeless in shelter (Lehman 1997) (follow up: 12 months)												
1	Randomised trials	Very serious [a]	Not serious	Not serious	Serious [b]	none	77	75	-	MD **6.2 fewer** (9.5 fewer to 2.89 fewer)	⊕◯◯◯ VERY LOW	CRITICAL
Number of days in community housing (Lehman 1997) (follow up: 12 months)												
1	Randomised trials	Very serious [a]	Not serious	Not serious	Serious [b]	none	77	75	-	MD **50.1 more** (46.15 more to 54.04 more)	⊕◯◯◯ VERY LOW	CRITICAL
Number of days in stable housing in previous month (Morse 2006) (Follow-up: 24 months)												
1	Randomised trials	Serious [d]	Not serious	Not serious	Serious [b]	none	54	49	-	MD **5.7 days more** (0.59 more to 10.8 more)	⊕⊕◯◯ LOW	CRITICAL
**Outcome: Mental Health**												
Mental health score assessed with SF-36 (Lehman 1997) (follow-up: 6 months)												

1	Randomised trials	Very serious [a]	Not serious	Not serious	Serious [b]	none	77	75	-	MD **6 higher** (0.73 higher to 11.26 higher)	⊕◯◯◯ VERY LOW	CRITICAL
Psychiatric symptoms using the Brief Psychiatric Rating Scale (BPRS) (Morse 2006) (Follow-up: 24 months)											
1	Randomised trials	Serious [d]	Not serious	Not serious	Serious [b]	none	54	49	-	MD **0.03 higher** (0.17 lower to 0.23 higher)	⊕⊕◯◯ LOW	CRITICAL
**Outcome: Quality of life**											
Overall quality of life using Lehman's QOLI (Essock 1998) Follow-up: 18 months											
1	Randomised trials	serious	not serious	not serious	serious [D]	none	131	131	The difference between ACT and SCM clients on the components of quality of life all favored the ACT condition, with a difference of one-half point or less, and three components reaches statistical significance [no further details]		⊕⊕◯◯ LOW	CRITICAL
**Outcome: Substance use**											
Ounces of alcohol consumption per week (Morse 1992) (follow-up: 12 months)											
1	Randomised trials	Serious [c]	Not serious	Not serious	Serious [b]	none	52	62	-	MD **1.88 higher** (0.64 lower to 4.4 higher)	⊕⊕◯◯ LOW	IMPORTANT
**Outcome: Hospitalization**											
Mean number of emergency department visits (Lehman 1997) (Follow-up: 12 months)											
1	Randomised trials	Very serious [a]	Not serious	Not serious	Serious [b]	none	77	75	-	MD **1.2 fewer** (2.03 fewer to 0.36 fewer)	⊕◯◯◯ VERY LOW	IMPORTANT

CI: Confidence interval; **SMD:** Standardised mean difference; **OR:** Odds ratio; **MD:** Mean difference

Explanations

a. High risk of performance bias, attrition bias and reporting bias. Unclear risk of selection bias and detection bias.
b. Small sample size (<300 participants)
c. Unclear risk of selection bias (random sequence generation and allocation concealment), high risk of performance bias (blinding), and unclear risk of detection bias (outcome assessment)
d. High risk of performance bias, unclear risk of selection and detection bias

Appendix 5.4: CTI GRADE Evidence Profile

Certainty assessment							№ of patients		Effect		Certainty	Importance
№ of studies	Study design	Risk of bias	Inconsistency	Indirectness	Imprecision	Other considerations	Case Management	Comparison	Relative (95% CI)	Absolute (95% CI)		
**Outcome: Housing Stability**												
Odds of homelessness at the end of the follow-up period (the final three six-week intervals) (Herman 2011) (follow up: 18 months)												
1	randomised trials	serious [a]	not serious	not serious	Serious [b]	none	3/58 (5.2%)	11/59 (18.6%)	**OR 0.22** (0.06 to 0.88)	**138 fewer per 1,000** (from 173 fewer to 19 fewer)	⊕⊕◯◯ LOW	CRITICAL
Mean number of: days rehoused (De Vet 2017) (follow up: 9 months)												
1	randomised trials	serious [c]	not serious	not serious	Serious [d]	none	80	82	-	MD **8.29 fewer** (22.79 fewer to 6.21 more)	⊕⊕◯◯ LOW	CRITICAL
Percentage of participants who were homeless at the last month of trial (Susser 1997) (follow up: 18 months)												
1	randomised trials	serious [e]	not serious	not serious	Serious [b]	none	4/48 (8.3%)	11/48 (22.9%)	**OR 0.30** (0.08 to 1.04)	**147 fewer per 1,000** (from 206 fewer to 7 more)	⊕⊕◯◯ LOW	CRITICAL
Odds of extended homelessness - more than 54 nights (Susser 1997) (follow up: 18 months)												
1	randomised trials	serious [e]	not serious	not serious	Serious [b]	none	10/48 (20.8%)	19/48 (39.6%)	**OR 0.40** (0.16 to 0.99)	**188 fewer per 1,000** (from 301 fewer to 2 fewer)	⊕⊕◯◯ LOW	CRITICAL
**Outcome: Mental Health**												
Psychological symptoms measured as BSI Global Severity Index (De Vet 2017) (follow up: 9 months)												
**1**	randomised trials	serious [c]	not serious	not serious	Serious [d]	none	85	77	-	**MD 0.13 lower** (0.29 lower to 0.03 higher)	⊕⊕◯◯ LOW	CRITICAL
**Outcome: Quality of Life**												
Quality of life using Lehman's Brief Quality of Life Interview (De Vet 2017) (follow up: 9 months)												

1	randomised trials	serious [c]	not serious	not serious	Serious [d]	none	90	83	-	**MD 0.18 higher** (0.2 lower to 0.56 higher)	⨁⨁◯◯ LOW	CRITICAL
**Outcome: Substance use**												
Excessive (5+ drinks/day) use of alcohol in a 30 day period; assessed with the European Addiction Severity Index (De Vet 2017) (follow-up: 9 months)												
1	randomised trials	serious [c]	not serious	not serious	Serious [b]	none	19/87 (21.8%)	21/80 (26.3%)	**OR 0.78** (0.38 to 1.59)	**45 fewer per 1,000 (from 143 fewer to 99 more)**	⨁⨁◯◯ LOW	IMPORTANT
Use of cannabis In a 30 day period; assessed with the European Addiction Severity Index (De Vet 2017) (follow-up: 9 months)												
1	randomised trials	serious [c]	not serious	not serious	Serious [b]	none	13/87 (14.9%)	18/80 (22.5%)	**OR 0.60** (0.27 to 1.33)	**77 fewer per 1,000 (from 152 fewer to 54 more)**	⨁⨁◯◯ LOW	IMPORTANT
**Outcome: Hospitalization**												
Psychiatric rehospitalization at the end of the follow-up period (the final three six-week intervals) (Tomita 2012) (follow-up: 18 months)												
1	Randomised trials	Serious [f]	Not serious	Not serious	Serious [b]	none	NR	NR	**OR 0.11** (0.01 to 0.96)	Not estimable	⨁⨁◯◯ LOW	IMPORTANT
**Outcome: Income**												
Earned income in the first nine months (Jones 2003) (follow up: 9 months)												
1	randomised trials	serious [e]	not serious	not serious	Serious [d]	none	47	44	-	MD **85 more** (110.44 fewer to 280.44 more)	⨁⨁◯◯ LOW	IMPORTANT
Earned income in the second nine months (Jones 2003) (follow up: 18 months)												
1	randomised trials	serious [e]	not serious	not serious	Serious [d]	none	47	44	-	**MD 27 more** (198.36 fewer to 252.36 more)	⨁⨁◯◯ LOW	IMPORTANT

CI: Confidence interval; **SMD:** Standardised mean difference; **OR:** Odds ratio; **MD:** Mean difference, **NR:** Not reported

Explanations

a. . High risk of selection bias and blinding of participants and personnel
b. Less than 100 events
c. High risk of blinding of participants and personnel (performance bias), and high risk of blinding of outcome assessment (detection bias)
d. Small sample size (<300 participants)
e. Unclear risk of selection and high risk of performance bias.
f. High risk of selection bias and performance bias
