# Supplementary material for: The effectiveness of case management interventions for the homeless, vulnerably housed and persons with lived experience: A systematic review
Source: PLoS One. 2020 Apr 9;15(4):e0230896. doi: 10.1371/journal.pone.0230896 (PMC7313544; doi:10.1371/journal.pone.0230896)

Appendix S7: Fixed effects and random effects meta-analyses

FIXED EFFECT MODEL:

	ICM			Usual care				Std. Mean Difference
**Study or Subgroup**	**Mean**	**SD**	**Total**	**Mean**	**SD**	**Total**	**Weight**	**IV, Fixed, 95% CI**
Cox 1998	10.7	19.7	108	19.2	23.7	83	28.0%	-0.39 [-0.68, -0.10]
Grace 2014	64	117	196	77	135	174	56.1%	-0.10 [-0.31, 0.10]
Toro 1997	3.4	15.1	54	10	37.9	51	15.9%	-0.23 [-0.61, 0.15]
**Total (95% CI)**			**358**			**308**	**100.0%**	**-0.20 [-0.36, -0.05]**

Heterogeneity: Chi² = 2.60, df = 2 (P = 0.27); I² = 23%
Test for overall effect: Z = 2.62 (P = 0.009)

Std. Mean Difference
IV, Fixed, 95% CI

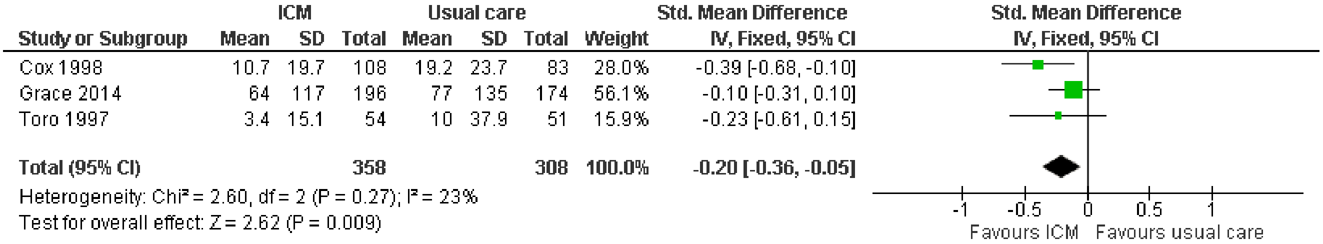

	ICM			Usual care				Std. Mean Difference
**Study or Subgroup**	**Mean**	**SD**	**Total**	**Mean**	**SD**	**Total**	**Weight**	**IV, Fixed, 95% CI**
Cox 1998	25.4	26	108	21.7	26.4	83	33.7%	0.14 [-0.15, 0.43]
Grace 2014	159	154	196	168	159	174	66.3%	-0.06 [-0.26, 0.15]
**Total (95% CI)**			**304**			**257**	**100.0%**	**0.01 [-0.16, 0.18]**

Heterogeneity: Chi² = 1.22, df = 1 (P = 0.27); I² = 18%
Test for overall effect: Z = 0.11 (P = 0.91)

Std. Mean Difference
IV, Fixed, 95% CI

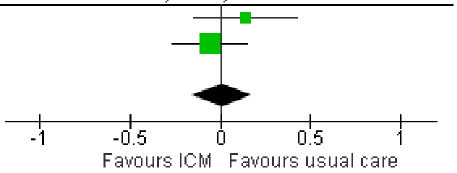

RANDOM EFFECT MODEL:

	ICM			Usual care				Std. Mean Difference
**Study or Subgroup**	**Mean**	**SD**	**Total**	**Mean**	**SD**	**Total**	**Weight**	**IV, Random, 95% CI**
Cox 1998	25.4	26	108	21.7	26.4	83	36.6%	0.14 [-0.15, 0.43]
Grace 2014	159	154	196	168	159	174	63.4%	-0.06 [-0.26, 0.15]
**Total (95% CI)**			**304**			**257**	**100.0%**	**0.02 [-0.17, 0.20]**

Heterogeneity: Tau² = 0.00; Chi² = 1.22, df = 1 (P = 0.27); I² = 18%
Test for overall effect: Z = 0.16 (P = 0.87)

Std. Mean Difference
IV, Random, 95% CI

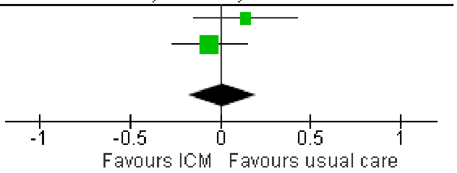

	ICM			Usual care				Std. Mean Difference
**Study or Subgroup**	**Mean**	**SD**	**Total**	**Mean**	**SD**	**Total**	**Weight**	**IV, Random, 95% CI**
Cox 1998	10.7	19.7	108	19.2	23.7	83	30.7%	-0.39 [-0.68, -0.10]
Grace 2014	64	117	196	77	135	174	50.1%	-0.10 [-0.31, 0.10]
Toro 1997	3.4	15.1	54	10	37.9	51	19.2%	-0.23 [-0.61, 0.15]
**Total (95% CI)**			**358**			**308**	**100.0%**	**-0.22 [-0.40, -0.03]**

Heterogeneity: Tau² = 0.01; Chi² = 2.60, df = 2 (P = 0.27); I² = 23%
Test for overall effect: Z = 2.33 (P = 0.02)

Std. Mean Difference
IV, Random, 95% CI

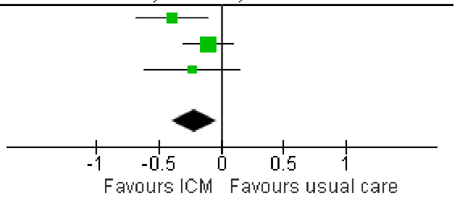

Supplement: S7 File — (PDF) [file pone.0230896.s007.pdf]
