# Supplementary material for: The effectiveness of case management interventions for the homeless, vulnerably housed and persons with lived experience: A systematic review
Source: PLoS One. 2020 Apr 9;15(4):e0230896. doi: 10.1371/journal.pone.0230896 (PMC7313544; doi:10.1371/journal.pone.0230896)
Supplement: Attachment — Submitted filename: Revision 1 - Ponka et al. - Response to reviewers Nov 2019.docx [file pone.0230896.s008.docx]

**Kevin Pottie, MD CCFP MCISc FCFP**
85 Primrose Ave, room 307, Ottawa, ON
[kpottie@uottawa.ca](mailto:kpottie@uottawa.ca)

**Re:** The Effectiveness of Case-Management Interventions for the Homeless, Vulnerably Housed and Persons with Lived Experience: A Systematic Review and Meta-Analysis (reference number PONE-D-19-19380)

**Response to editors and peer-reviewers**

**Editorial comments**

Please ensure that your manuscript meets PLOS ONE's style requirements, including those for file naming

Response: Thank you. We have reviewed PLOS ONE’s style requirements and have edited our manuscript as necessary.

Please upload a copy of Figures 3 &4, to which you refer in your text on page 36. If the figure is no longer to be included as part of the submission please remove all reference to it within the text.

Response: Figures 3 and 4 are the forest plots representing our pooled meta-analyses. We have uploaded them separately as per your request.

**Peer reviewers’ comments**

Reviewer #1

This is a review article on case management interventions for persons who are homeless or otherwise unstably housed, focused on ACT, ICM, and CTI. The effects of these interventions on health and social outcomes are discussed, as well as the quality of the evidence surrounding each intervention. These interventions are critical for improving care for a very vulnerable population and a systematic evaluation of the evidence surrounding these practices is valuable. The article is well-written. Some specific comments are offered below.

Response: Thank you for taking the time to review our manuscript and highlight the importance of such a comprehensive review of the literature on the effectiveness and cost effectiveness of case management interventions among homeless and vulnerably housed populations. We have addressed your comments and feedback. Kindly see our responses below.

Abstract

• The conclusions of the abstract reflect the need to balance fidelity to an intervention’s components (used to achieve outcomes shown in research settings) and adaptation to meet the real-world context of under-resourced settings. To that end, might be good to add a sentence to the background of the abstract highlighting the clinical relevance of this review, i.e., what is the utility of studying these case management approaches with regards to real world care.

Response: Thank you for this comment. The definitions of case management models in the very heterogeneous literature makes this addition very relevant. This also links to your comment below about SCM which we address further. In fact, our conclusions reflect the need to better understand the link between intensity and effect, especially in complex and variable primary care settings.

• There seems to be a comparison between mainstream case management and three more intensive CM models, but mainstream CM is not mentioned in the abstract

Response: Thank you for highlighting this discrepancy. We have added to our abstract findings of mainstream case management

Introduction

• The first sentence references structural challenges (of which there are many) - however, the second sentence describes individual level factors that are barriers to care, as opposed to structural challenges.

Response: Thank you, we have amended these sentences to improve clarity.

• Table 1

o SCM - is this limited to persons engaging in primary care? I think that routine case management happens for many homeless individuals who receive social services but who do not receive health care services in primary care settings. A concern for throughout the manuscript is that SCM is very challenging to define and very diverse across settings and studies.

Response: Thank you for this comment. We agree that SCM is very heterogeneous. We have added additional remarks to that effect on our introduction. SCM is also not limited to persons engaging in primary care, although it is common. We have revised Table 1 to reflect that SCM may be delivered to persons with complex care needs.

o CTI - generally this model is not just with any transition, but the transition between an institutional setting to community living

Response: We sought evidence on critical time interventions that aimed to help individuals transition from a state of precarious housing and into more stable accommodation. As a result of our search, we found evidence on three CTI trials that assist individuals transition from shelters to more stable housing as well as one trial devising a transition plan after discharge from the hospital. Thank you for you comment. We will make this more clear in Table 1 and the results section.

• Interested to hear more about how the Delphi consensus panel helped prioritize interventions of interest - were SCM, ICM, CTI, ACT selected because of this panel? A few sentences about this in the methods as opposed to the intro might be helpful.

Response: Thank you for this comment. We have provided additional details in the methods (see section “Selection of priority interventions”) regarding the Delphi process and the rationale for choosing these models of case management.

Results

• The Hurlbut study (SCM) is a HUD-VASH study, which is a PSH program (I thought these were excluded to be part of a separate review)

Response: Thank you. Even though the Hurlburt study is part of the HUD-VASH project. The findings of the two publications reported on the added benefits of providing case management to participants regardless of their housing arrangements. We sought evidence on every study that reported on the benefits (or harms, if found) of case management interventions, and thus we have included these publications for that purpose.

• Clark et al (2003) - the comprehensive housing program sounds very much like PSH

Response: You are correct. The comprehensive housing program is very much a replication of PSH. However, participants allocated to the other arm of this trial are provided with case management services only. We included this study in our analysis to assess whether case management only was superior or equivalent to PSH in improving our outcomes of interest.

• The findings on ACT - findings on hospitalization were mostly positive, which I think is consistent with the literature. How does this work juxtaposed with the assertion that benefits of ACT on mental health outcomes were minimal - hospitalization is generally a proxy for mental health outcomes.

Response: We agree and have rephrase the sentence to reflect the reasonably positive effects of ACT interventions in reducing in psychiatric symptoms across studies.

• Cost effectiveness: how could total cost incurred by SCM clients be greater than total cost for ACT clients?

Response: Thank you for your comments. The total cost of SCM was higher than ACT in the case when the studies included all cost components incurred to society (termed the ‘societal perspective’). Specifically, the slightly higher cost of SCM was due to the fact that SCM clients had nominally more frequent visits to outpatient health care and other community services, more arrest episodes, and incurred higher family time costs compared to ACT clients. We have clarified this point in the cost and cost-effectiveness section.

Discussion

• Appreciate the recognition at the top of the discussion that the interventions, populations, and outcomes were heterogeneous, which makes it hard to analyze the data - it would be good to have this sort of statement up front at the end of the introduction statement

Response: We agree, and have provided two sentences at the end of the introduction to highlight the heterogeneity of the interventions at hand.

• A fundamental question is if SCM is good for a different subgroup of homeless people than the more intensive interventions studied. Or, is SCM just too low intensity for homeless people, but acceptable for a less vulnerable population? The article basically says that more intensive case management is needed for everyone in this population - I’m not sure that is true, as there has got to be some match between clinical acuity and the acuity of the CM intervention needed; SCM is also such a broad and vague term encompassing many different things in studies and in real-world settings

Response: Thank you very much for these thoughtful questions. We agree that the definition of SCM is challenging and thus decided to take a broad approach. We attempted to distinguish the differences in CM in Table 1. However, comparing SCM to more intensive models was not the focus of the review, and indeed such a comparison would likely be based on comparable populations - per Table 1, the more intensive approaches in fact aimed at specific subsets of homeless popullations. Because of all these factors, not to mention that issues of fidelity that you raise, we tempered the strength of our conclusions.

• There is a statement in the discussion that persons with lived experience were integrated on the research team - not clear how this took place or in what capacity they were involved in these analyses

Response: Thank you for highlighting the shortage in describing the role of our people with lived experience of homelessness in this project. We have provided more information regarding this issue in the methods section.

Reviewer #2: Thank you for the opportunity to review this interesting manuscript. Since I am not an expert in the topic, my comments will focus on the systematic review and meta-analysis methodology and reporting.

Response: Thank you for reviewing our work and providing feedback on our methods. An important strength of our review is the rigorous methodology we have used in screening, data collection and management, data analysis, and reporting processes. We have addressed all your comments. Kindly find our responses below.

Major Concern:

The results of this systematic review and meta-analysis should focus more on the meta-analytic findings. Nearly all of the results presented resort to counting p-values or describing the findings from specific studies. The beauty of meta-analysis is to move beyond p-counting so that you can show the overall effect of a type of intervention. Of the 56 studies in this review, I’m only seeing 3 studies meta-analyzed, for only two outcomes. No rationale is provided for why a meta-analysis was not performed on any other outcomes.

Response: Thanks, meta-analysis is one of the most robust statistical methods to synthesize outcome data to provide a quantitative estimate and we used this method whenever outcome data permitted. We attempted to meta-analyze all outcomes, but heterogeneity in interventions, outcomes and time-points precluded this for the majority of outcomes. We have clarified this in the methods section.

Even the narrative review results are rudimentary, with very little synthesis reported.

Response: Thank you. It appears we did not clearly describe our synthesis. Given that the majority of our synthesis is narrative, we have referred to the SWiM (Synthesis Without Meta-Analysis) reporting guidelines (previously named “Improving the Conduct and reporting of Narrative Synthesis of Quantitative data (ICONS-Quant)”), which is presently under consideration for publication (must be kept confidential). The ICONS-Quant items are intended to complement PRISMA. ICONS-Quant relates to the methods and reporting of narrative synthesis, while PRISMA relates to the entire systematic review process. We have modified our paper to reflect these reporting items.

Additional comments:

1. The protocol has not been published, so reviewers only have the limited methods section to use to evaluate the review methods. While the methods appear to be rigorous, more detail would be helpful, particularly in the data analysis section. Also, the author guidelines for PLOS One state that systematic reviews without published protocols should include the protocol in the supplementary material.

Response: Thank you for this comment. Our protocol was published during the peer-review period. We have updated our reference list and provide a DOI to the open-access publication for reviewers to consider. Please see: <https://doi.org/10.1002/cl2.1048>

2. Was the protocol registered with a systematic review registry, such as PROSPERO? I did not see a PROSPERO registration number mentioned. PLOS One submission guidelines require the protocol registry number to be included in the abstract.

Response: Thank you for this question. We registered our title (2018) and protocol (2019) with the Campbell Collaboration, which is considered a systematic review registry. We have made this more explicit in our manuscript under “Methods”. We have updated our reference list and provide a DOI to the open-access publication for reviewers to consider.

3. Please address in the methods how you managed studies that had multiple interventions using the same control group in your meta-analyses.

Response: Thank you for this comment. We have added the following to our methods section to clarify this point: To prevent double-counting of outcomes, individual records were carefully screened to identify unique trial studies. Each study was then evaluated for potential overlap using study design, enrollment and data collection dates, authors and their associated affiliations and the reported selection and eligibility criteria in the studies to inform the assessment. Studies deemed to be at risk for double-counting were discussed by the research team and decisions for inclusion in meta-analysis (and any additional analyses) were made.

4. The PRISMA flow diagram should include a summary of the reasons for exclusion for the 214 studies excluded at the full-text review stage in the original search. Don’t just make readers go to the supplementary files and then expect the reader to count the reasons themselves. While I admire the level of detail you’re including in your tables and supplementary files, the whole point of a review is to summarize and synthesize for readers.

Response: Thank you for this comment. We have updates this figure with the following information:

Excluded studies n=214

Reasons:

Wrong study design n=51

Irrelevant outcomes n=8

Wrong population n=25

Wrong intervention n=117

Wrong publication type n=~~117~~ 10

Could not be retrieved n=3

5. Table 9 needs a legend that defines the abbreviations for the intervention types.

Response: Thank you, we have added a legend to this table which defines the abbreviations.

6. On p. 35, line 189, the word “trivial” may not be the best word choice, as it can imply a value judgement. Perhaps something like “equivocal” would be better?

Response: Thank you for this suggestion, we have made the appropriate changes.

7. Are Figures 1 and 2 data from just one study? This is not clear from the figures alone, as there is no study cited, and forest plots typically imply meta-analytic results across studies.

Response: Thank you, these figures have been removed.

8. The figures on page 36 are not labeled.

Response: Thank you, we have received feedback against using these forest plots in our results section as they come from the same study, and therefore, we have decided to replace them with a descriptive synthesis of evidence from these studies.

Reviewer #3: Overall, this is a well-written analysis of case management interventions of homeless or vulnerably-housed individuals. This is not my field of study, so I cannot comment on the relevance of this review to this field or the quality of the qualitative analysis portion, other than to say that it was thorough. However, I have some concerns regarding the meta-analysis. It seems improper to use a random-effects meta-analysis in a meta-analysis including only 2 or 3 studies. My suspicion is that, in practice, your conclusion that ICM reduces number of days homeless will be robust to either approach: fixed effects vs. random effects, but it would be encouraging to know that the random effects approach has not under-estimated the confidence intervals on the pooled effect. I would recommend repeating the analyses from the figures on page 36 using a fixed effects approach (basically an inverse-variance weighted average of the studies) and indicate whether the results were similar or not to the random effects results.

Response: Thank you for this comment. As per your suggestion, we have rerun the analysis using both a fixed effects model and found that our conclusions were unchanged:

**FIXED EFFECT MODEL:**

**RANDOM EFFECT MODEL:**

However, we stand behind our original decision to publish the meta-analysis using a random effects model due to its consideration of heterogeneity. Under any interpretation, a fixed-effect meta-analysis ignores heterogeneity. In the meta-analyzed studies of our review, we have conceptual heterogeneity in populations (e.g. youth [Grace 2014] vs. mentally ill adults with children [Toro 1997] vs. chronic inebriated adults [Cox 1998]) and interventions ( time-limited ICM [Grace 2014] vs. ICM with job training [Toro 1997] vs long-term ICM [Cox 1998]). Given this conceptual heterogeneity, we could not assume that the true effect of intervention (in both magnitude and direction) is the same value in every study (i.e. fixed across studies). Instead of assuming that the intervention effects are the same, we assume that they follow (usually) a normal distribution. The assumption implies that the observed differences among study results are due to a combination of the play of chance and some genuine variation in the intervention effects.

Minor comments:

Tables 4-8 should go into a supplemental material file. This is a thorough summary of all of these studies and way too much information for the text of the manuscript.

Response: Thank you for this suggestion, we have moved these tables to supplemental material (S4).

Figures 1 and 2: Are these from a single study? If so, I don't understand why it's necessary to present these findings that have been presented elsewhere. I would recommend dropping these figures and succinctly summarizing the results in the text.

Response: Thank you for this suggestion, we have removed these figures.
