# Supplementary material for: The effectiveness of case management interventions for the homeless, vulnerably housed and persons with lived experience: A systematic review
Source: PLoS One. 2020 Apr 9;15(4):e0230896. doi: 10.1371/journal.pone.0230896 (PMC7313544; doi:10.1371/journal.pone.0230896)
Supplement: Attachment — Submitted filename: Revision 2 - Ponka et al. - Response to Reviewers.docx [file pone.0230896.s009.docx]

**Ponka et al. Case Management Review (Revision 2) Response to Reviewers	Feb 2020**

**Reviewer #1**: The authors sufficiently addressed the concerns I mentioned in the first round of reviews. The only thing missing from the discussion section at this point from my perspective is a more nuanced reflection that homeless people have different levels of acuity and thus have different levels of need for case management intensity. That is, ACT isn't for everyone, ICM isn't for everyone, these are case management models needed for certain people with certain functional limitations and there should be some effort to match needs to services.

**Response:** Thank you for reviewing our manuscript and for your helpful feedback. We agree that not all models of case management are appropriate for every individual, and that the services provided should be matched to client needs. We have edited our discussion section and have included the following: “*However, it is likely that a dose-response relationship may explain some of our findings, and that as higher intensity interventions such as ACT and ICM are more precisely defined, there may be greater attention to fidelity in their implementation (19). Alternatively, it is possible that lower intensity models work predominantly for homeless populations with less acute issues (or for those that are precariously housed), and this would suggest the importance of matching the intensity of the intervention with the acuity of need*.”

**Reviewer #2**: I’d like to thank the authors for their response to the reviewer comments. I understand how challenging it is to do a project of this scope.

**Response:** Thank you for your support.

The authors’ response to the critique about registering the systematic review is somewhat misleading. Publishing a review protocol is not the same as registering the protocol. Submitting the title to the Campbell Collaboration is not the same as listing the planned review in a publicly accessible registry prior to conducting the review. Further, the authors’ wording on p. 5 (lines 86-87) may mislead readers to think that this review was done under the auspices or sponsorship of the Campbell Collaboration (“…published by the Campbell Collaboration”), but my reading of the review protocol is that this was not the case. It might be simpler and more accurate to state that the review protocol was not registered, but was published in 2019 (and then cite the protocol paper).

**Response:** Thank you for this feedback. You are correct; the protocol for this review was not registered in a publicly available registry and we agree that the original reporting of this in our manuscript may be misleading. We have edited this paragraph of the methods section to be explicit about this and provide an explicit statement for our reader: “*We conducted a systematic review according to a published peer-reviewed protocol (29). The protocol was not registered in an open-access registry (e.g. PROSPERO) prior to publication*”. Thank you for bringing this to our attention, this is an important lesson learned for the future.

I still think that the authors’ narrative synthesis of results is rather rudimentary, but I understand that it can be difficult to write a narrative synthesis without falling into the trap of describing things study-by-study.

**Response:** Thank you for this comment. Despite frequent use and longstanding concerns about the validity of narrative synthesis, there has been almost no methodological development to promote clearer methods for narrative synthesis, despite its use in nearly half of health related systematic reviews. We have attempted to tabulate meaningful results (Table 4), a data presentation method suggested by the SWiM reporting guidelines, but we do recognize the limited richness of this synthesis. We appreciate your understanding.

I’d like to put in a note of support for the authors’ response to the first comment from Reviewer 3. I agree with the authors that a random effects model is the appropriate approach for meta-analyzing these types of studies, for the very reasons they give in their rationale.

**Response:** Thank you very much for this support. We have worked hard to achieve methodological rigor in our review and are confident in this decision.

Ultimately, however, this manuscript is kind of a bait-and-switch. The title tempts a reader with a meta-analysis, but then out of the 56 studies included, only 3 studies were used for two (related) outcomes for only one of the types of case management. I have a genuine concern that the narrative review findings from this manuscript will be cited as though they are based on a meta-analysis when in fact they aren’t. While I don’t want to take away from the authors the work they did on the small meta-analysis in this paper, I also think that it might be more honest to remove “and Meta-analysis” from the title, since the overwhelming majority of this review’s findings are not based on meta-analysis.

**Response:** This is an excellent point. We agree and have removed “and meta-analysis” from our title.

**Reviewer #3:** Regarding the fixed effects vs. random effects issue, the problem isn't that you don't have heterogeneity. The problem is that it's hard to quantify the heterogeneity with only 2-3 studies. The fact that the fixed effects analysis has qualitatively similar results should be briefly mentioned in the text and the results should be added to supplemental information.

**Response:** Thank you, this is a good idea. We have added a sentence to our methods section stating that we ran these parallel analyses, and have written in the results section that findings were unchanged (i.e. not dependent on fixed vs. random effects models). We have included these results in Appendix S7. We appreciate your constructive feedback.
